# SHARK‐capture identifies functional motifs in intrinsically disordered protein regions

**DOI:** 10.1002/pro.70091

**Published:** 2025-03-18

**Authors:** Chi Fung Willis Chow, Swantje Lenz, Maxim Scheremetjew, Soumyadeep Ghosh, Doris Richter, Ceciel Jegers, Alexander von Appen, Simon Alberti, Agnes Toth‐Petroczy

**Affiliations:** ^1^ Max Planck Institute of Molecular Cell Biology and Genetics Dresden Germany; ^2^ Center for Systems Biology Dresden Dresden Germany; ^3^ Cluster of Excellence Physics of Life Technische Universität Dresden Dresden Germany; ^4^ Biotechnology Center (BIOTEC), Center for Molecular and Cellular Bioengineering Technische Universität Dresden Dresden Germany

**Keywords:** alignment‐free, IDRs, motif detection, sequence‐to‐function

## Abstract

Increasing insights into how sequence motifs in intrinsically disordered regions (IDRs) provide functions underscore the need for systematic motif detection. Contrary to structured regions where motifs can be readily identified from sequence alignments, the rapid evolution of IDRs limits the usage of alignment‐based tools in reliably detecting motifs within. Here, we developed SHARK‐capture, an alignment‐free motif detection tool designed for difficult‐to‐align regions. SHARK‐capture innovates on word‐based methods by flexibly incorporating amino acid physicochemistry to assess motif similarity without requiring rigid definitions of equivalency groups. SHARK‐capture offers consistently strong performance in a systematic benchmark, with superior residue‐level performance. SHARK‐capture identified known functional motifs across orthologs of the microtubule‐associated zinc finger protein BuGZ. We also identified a short motif in the IDR of *S. cerevisiae* RNA helicase Ded1p, which we experimentally verified to be capable of promoting ATPase activity. Our improved performance allows us to systematically calculate 10,889 motifs for 2695 yeast IDRs and provide it as a resource. SHARK‐capture offers the most precise tool yet for the systematic identification of conserved regions in IDRs and is freely available as a Python package (https://pypi.org/project/bio-shark/) and on https://git.mpi-cbg.de/tothpetroczylab/shark.

## 1 INTRODUCTION

Intrinsically disordered regions (IDRs) have garnered significant attention, resulting in mounting evidence demonstrating their wide repertoire of functions. Due to their inherent flexibility, multi‐valency, and ability to sample multiple conformations, IDRs are adept at a wide array of binding‐related functions, including molecular recognition, protein modification, and molecular assembly formation (Fuxreiter et al., 2014). Such binding activities vary across biological processes, ranging from transient binding to enzymes to mediate post‐translational modifications (Evans et al., 2022; Jumper et al., 2021) to the interactions of “scaffold” IDRs that mediate phase separation leading to the formation of biomolecular condensates (Bryant et al., 2022). Moreover, despite advances in the structural prediction of protein interactions, such as AlphaFold2 and AlphaFold‐multimer (Bryant et al., 2022; Evans et al., 2022; Jumper et al., 2021), the systematic determination of precise protein binding sites, particularly for IDRs, remains challenging. This is further exacerbated by the limitations of experimental techniques, as even higher throughput techniques, such as cross‐linking mass spectrometry (Lee & O'Reilly, 2023; Piersimoni et al., 2022), which can identify specific binding/interaction sites with residue‐level precision, are unable to delineate regions that may confer functional regulation. As such, the systematic detection of functionally critical regions of IDRs remains a key step toward a comprehensive understanding of the sequence‐function relationships in IDRs.

One such example is a class of 3–10 amino acid long peptides known as short linear motifs (SLiMs) (Davey et al., 2012). Otherwise known as minimotifs or linear motifs, SLiMs have been widely known to be a key functional feature of IDRs. To that end, extensive efforts have been made to identify functional SLiMs in IDRs, with the eukaryotic linear motif (ELM) database curating continued efforts to detect and verify these motifs (Kumar et al., 2022). Concomitantly, this has driven the development of a multitude of computational motif detection tools, of which MEME (Bailey et al., 2015), GLAM2 (Frith et al., 2008) and SLiMFinder (Davey et al., 2010) have been used for *de novo* motif detection (Christie et al., 2023; Martinez‐Goikoetxea & Lupas, 2023; Mihalič et al., 2023; Mumtaz et al., 2024; Presnell et al., 2024) and accordingly are consistently benchmarked against for SLiM detection (Kelil et al., 2017; Prytuliak et al., 2017). Each of these tools uses vastly different underlying principles for motif detection. MEME (Multiple Expectation–Maximization for Motif Elicitation) utilizes alignment‐free expectation maximization to detect un‐gapped motifs. SLiMFinder is also alignment‐free but instead relies on (exhaustive) enumeration to find variable‐length motifs and accepts wild‐card spacers where any residue is accepted. GLAM2 (Gapped Local Alignment of Motifs), on the other hand, is an alignment‐based tool to identify gapped motifs. Despite the availability and ongoing development of motif detection tools, however, systematic and accurate identification of SLiMs in IDRs remains challenging.

Much of the challenge can be attributed to the unique evolutionary properties of IDRs (Chow & Toth‐Petroczy, 2025). Despite the conservation of sequences critical for IDR functions, including SLiMs, IDRs are known to evolve more rapidly than ordered/structured regions (Brown et al., 2002; Khan et al., 2015; Tóth‐Petróczy & Tawfik, 2011), with a higher rate of substitutions and insertions/deletions (InDels) between homologs. Moreover, since IDRs are enriched in a particular set of amino acids (Pro, Arg, Gly, Gln, Ser, Lys, Ala, and Glu) (Van Der Lee et al., 2014), they are usually low in sequence complexity and repetitive (Campen et al., 2008; Jorda et al., 2010; Romero et al., 2001; Vacic et al., 2007; Williams et al., 2001). Altogether, this leads to greater sequence divergence between homologs, thereby rendering alignment inaccurate and ineffective in identifying conserved SLiMs within. Indeed, the rapid evolution of IDRs has been proposed to promote *ex nihilo* emergence (and loss) of SLiMs (Davey et al., 2015). Moreover, the extensive degeneracy in SLiMs, i.e. the allowance of multiple residues at a particular position, also poses a challenge for motif detection tools when assessing which putative SLiM sequences can be considered equivalent. The incorporation of amino acid “equivalency groups” has attempted to solve the issue, but this requires a rigid and pre‐defined classification of amino acids (Davey et al., 2010; Exarchos et al., 2011). The problem is further compounded by the fact that interactions involving IDRs are usually multivalent, i.e. using multiple sites (usually each of relatively low affinity) to achieve high avidity whilst maintaining specificity (Banani et al., 2017; Ditlev et al., 2018; Fung et al., 2018). Given that these interaction sites/motifs are interspersed along the sequence and can appear in various orders, the collinearity constraint of alignment further hampers the ability to detect all such motifs. Collectively, these challenges manifest in consistently poor SLiM detection performance both in terms of recall and precision, as indicated in previous studies (Prytuliak et al., 2017). This low recall of known SLiMs leads to an inability to accurately predict new SLiMs or other functional regions in IDRs, ultimately hampering our attempts to further understand the functional sequence space of IDRs. Moreover, even tools that offer relatively higher recall within the existing repertoire, such as GLAM2, often overpredict a large region, leading to very low precision (Figure 2f and Table 1). This is particularly troubling for experimental investigations, where the prediction of such extensive regions would result in large‐scale deletions/mutations that fail to pinpoint the exact functional region within. Altogether, alignment‐free innovations are required to further the field of motif detection, particularly in highly divergent IDRs.

**FIGURE 1:**
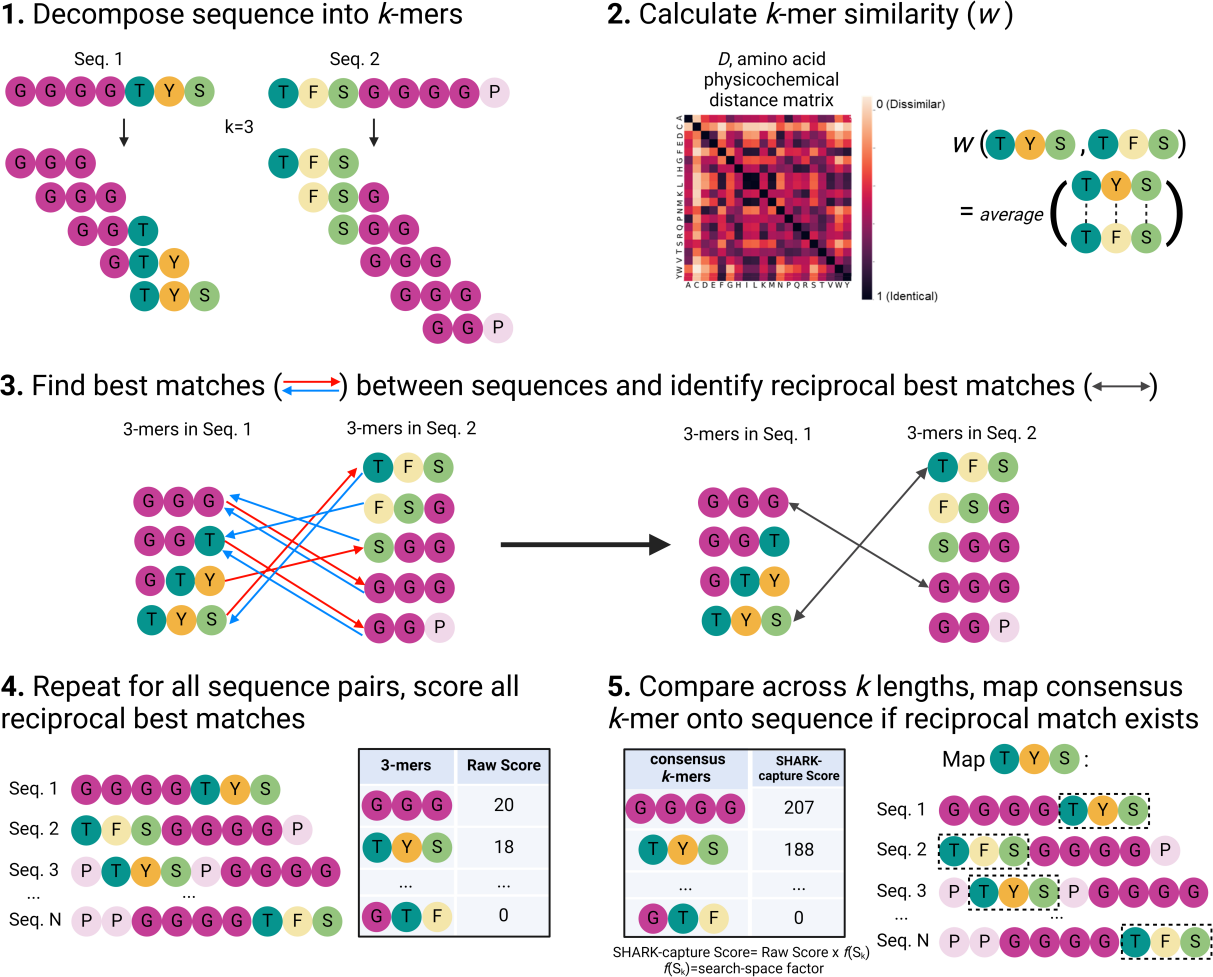
Overview of the SHARK‐capture algorithm. There are 5 main steps to the algorithm. (1) Decomposition of sequences into *k*‐mers. (2) Calculating *k*‐mer similarity. (3) Finding reciprocal best matches between the sequences. (4) Aggregating the results across all sequence pairs to identify highly conserved consensus *k*‐mers. (5) Finally, mapping highly conserved consensus *k*‐mers back onto the sequences.

**TABLE 1:** Performance of SHARK‐capture and other motif detection tools on the ELM benchmark dataset, 3679 SLiMs across 252 ELM classes (Data S1).

Tool	Site level			Residue level		
	Mean F1	Mean recall	Mean precision	Mean F1	Mean recall	Mean precision
GLAM2	*0.202*	0.191	*0.222*	0.080	*0.176*	0.060
MEME	0.057	0.050	0.084	0.034	0.045	0.035
SLiMFinder	0.151	0.133	0.196	0.127	0.105	*0.184*
SHARK‐capture	0.185	*0.193*	0.184	0.129	0.111	0.176
SHARK‐capture (extended)	0.187	*0.193*	0.186	*0.132*	0.118	0.174

*Note*: Highest value is italicized.

We present SHARK‐capture, an extension of the alignment‐free SHARK algorithm (Chow et al., 2024) to tackle the motif detection challenge. To ascertain its efficacy in systematic SLiM identification, we benchmarked SHARK‐capture alongside the aforementioned tools on a recent eukaryotic linear motif (ELM) database release, revealing that it achieved strong performance both in detecting SLiM sites across the set of proteins where it achieved best‐in‐class recall (0.193), as well as identifying the specific motif residues within each sequence where it achieved best‐in‐class overall performance (mean F1 = 0.129). We further applied SHARK‐capture to motif detection tasks in two sets of orthologous IDRs and report that it could not only recognize known motifs but was also capable of detecting sets of putative compositionally biased sites, interspersed along the sequence, which could promote phase separation activity. Moreover, it detected a short (4 amino acid) motif in *S. cerevisiae* RNA helicase Ded1p, which we experimentally validated to confer a significant modulatory effect, reducing the ATPase activity of Ded1p by 50% upon mutagenesis. Altogether, SHARK‐capture identifies functional motifs in IDRs and hard‐to‐align regions with high precision.

## 2 RESULTS

### 2.1 SHARK‐capture detects conserved motifs in an alignment‐free approach

SHARK‐capture is based on the SHARK (Similarity/Homology Assessment by Relating K‐mers) algorithm, which innovates upon existing word/*k*‐mer‐based algorithms by facilitating the assessment of *k*‐mer similarity (Figure 1) (Chow et al., 2024). This is achieved by comparing amino acids in physicochemical space, utilizing information encoded in amino acid physicochemical distance matrices. By default, SHARK‐capture uses the Grantham Distance Matrix (Grantham, 1974), which assesses amino acid pairwise similarity based on polarity, side chain chemical composition, and size, although the algorithm can use any distance matrix if other *a priori* knowledge of important amino acid physicochemical features is available. Next, by comparing the similarity between *k*‐mers (*w*, defined as the mean similarity between amino acids at each position), the best match in the other sequence (*w*_max_) for any given *k*‐mer can be found. These steps form the core of the SHARK algorithm.

SHARK‐capture is an extension of SHARK for motif detection (Figures 1 and S1). Since any *k*‐mer will have a best match in the other sequence, the key strategy used in SHARK‐capture is the identification of *reciprocal* best matches, where *k*‐mer A (in the first sequence) is the best match of *k*‐mer B (in the second sequence) and vice versa. Since SHARK‐capture assumes that linear motifs contain conserved physicochemical properties at each position, reciprocal *k*‐mer matches are of particular significance since they indicate a bilateral correspondence between the two *k*‐mers, thereby suggesting their conservation between the two sequences. This is then repeated across all unique pairs, whereby the conservation of a *k*‐mer (a potential motif) is reflected by the number of reciprocal hits involving the *k*‐mer, accordingly yielding a higher score. By default, SHARK‐capture considers a range of *k*‐mer lengths from *k* = 3 to *k* = 10, consistent with the lengths of most motifs (Davey et al., 2012; Maiti & De, 2022). To allow for comparison between scores of different *k*‐mer lengths, each score is scaled by a factor reflecting the number of unique *k*‐mers for a given *k*‐mer length to give the *SHARK‐capture score*. This enables a fairer assessment of *k*‐mer conservation across *k*‐mer lengths and allows the most conserved, top‐scoring *consensus k*‐*mers*, that is, potential motifs, to be identified. Finally, each consensus *k*‐mer is then mapped back to a sequence by identifying and locating its most frequent reciprocal match, if any, within the sequence.

In practice, for a given set of sequences (as a *FASTA file*), a *physicochemical distance matrix* of choice and a set of (*k*_min_, *k*_max_) values, SHARK‐capture returns three main outputs: (1) a table of the top *n* consensus *k*‐mers ranked by their corresponding SHARK‐capture scores, (2) a table for each consensus *k*‐mer showing the mapped *k*‐mer for each sequence as well as its start and end coordinates, and (3) a sequence logo and corresponding probability matrix for each consensus *k*‐mer. Importantly, the only required input parameters (that would significantly alter the output) are highlighted in italics to provide users with a simple‐to‐use tool that requires minimal parameter tuning and optimization steps. In addition, we also developed a post‐processing protocol, SHARK‐capture (extended), applied to SHARK‐capture to extend predicted regions, aimed at increasing motif sensitivity in large‐scale, proteome‐wide analyses at slight cost to speed and precision (see Methods in Section 4). This optional step confers additional versatility to SHARK‐capture to users depending on their focus on sensitivity or precision.

### 2.2 SHARK‐capture offers consistently high performance in systematic detection of known SLiMs

**FIGURE 2:**
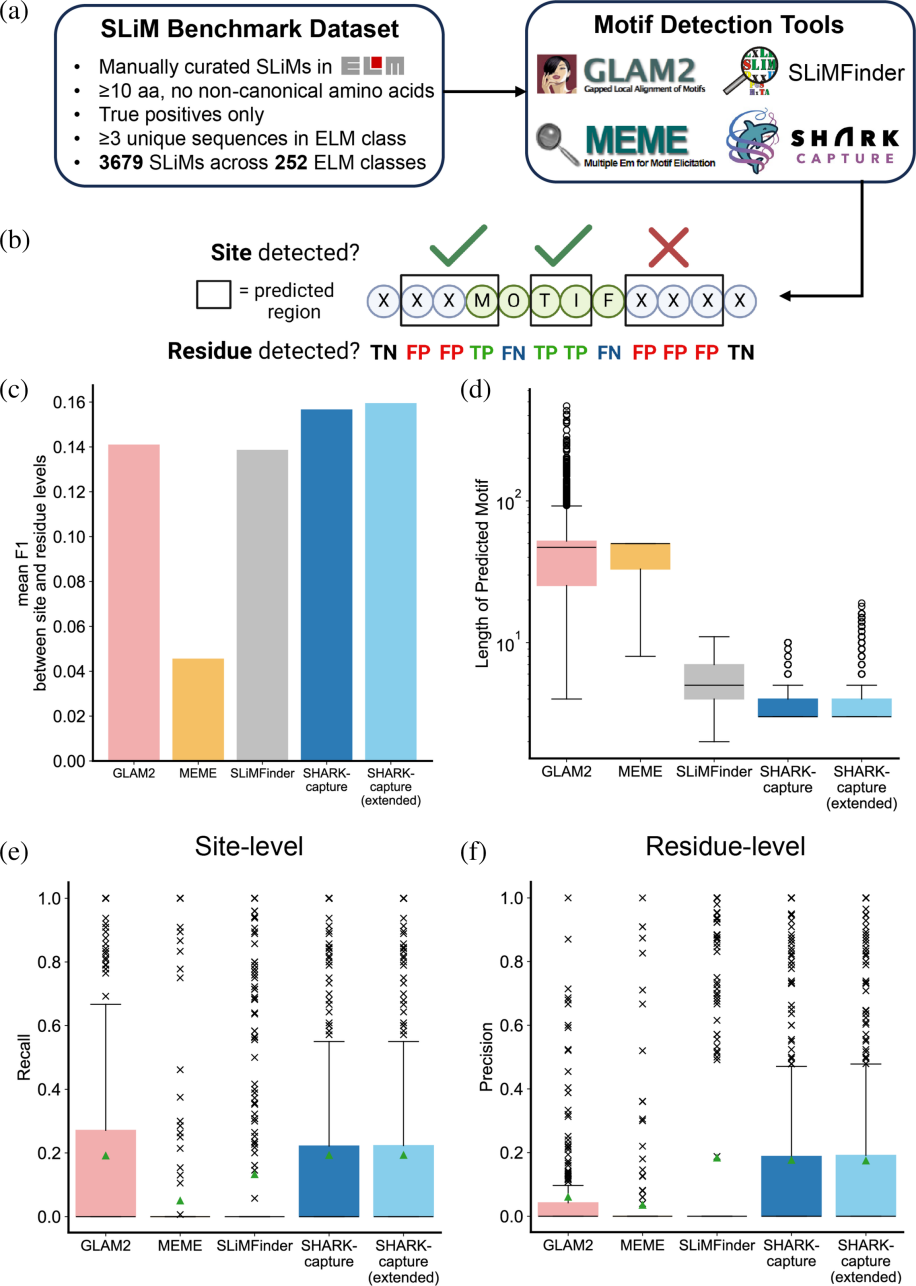
SHARK‐capture offers best‐in‐class performance in a systematic benchmark of SLiM detection. (a) Curation of SLiMs from the ELM database (Kumar et al., 2022) (http://elm.eu.org/). (b) Definition of detection success at site (top) and residue (bottom) levels. (c) SHARK‐capture outperforms existing tools, offering superior overall performance (F1) across both site and residue levels (averaged). (d) SHARK‐capture predicted motifs have a similar predicted motif length distribution to SLiMFinder, whereas GLAM2 and MEME generally find far longer motifs. Distribution of site‐level recall (e) and residue‐level precision (f) across 252 ELM classes (green triangle depicts mean).

To assess the performance of SHARK‐capture systematically as a motif detection tool, we curated a set of SLiMs from the eukaryotic linear motif (ELM) database (Kumar et al., 2022) (Figures 2a and S2) and benchmarked it against 3 popular motif discovery tools with different underlying methods: MEME, SLiMFinder, and GLAM2 (Figure 2a). We evaluated the precision, recall, and overall performance (F1 score) of the tools on each functional class of SLiMs (Figure S3), defined as an ELM class in the database, and report the unweighted mean precision, recall, and F1 metrics across all classes as overall indicators of tool performance (see Methods in Section 4).

In addition, we report these metrics at both site and residue levels, similar to Prytuliak *et al.* (2017). Site‐level performance assesses the ability of the tools to identify regions that at least partially contain the SLiM, prioritizing sensitive detection of SLiMs due to its leniency in accepting partial overlap. At the same time, the emphasis on sensitivity leads to a bias in favor of tools that predict longer regions. We therefore also highlight residue‐level performance, which focuses on prediction precision by evaluating the tool's ability to detect SLiM residues without including significant false positives, thereby penalizing tools that offer extensive over‐prediction of SLiM regions (see Methods in Section 4). The consideration of both site and residue level performance offers a sufficiently comprehensive assessment of tool performance (Figure 2b). We further apply a stringent criterion by only evaluating the top‐ranked SLiM, since each ELM class should only contain one unique conserved SLiM. We believe this represents a practical usage of a motif detection tool by an experimentalist, as we expect the top‐ranked SLiM will be the first choice to be experimentally validated in the absence of other *a priori* knowledge.

As summarized in Table 1, the benchmark results indicate the differing strengths and weaknesses of each tool but indicate the consistently favorable performance of SHARK‐capture in detecting ELMs. It achieved improved average F1 performance across site and residue levels with an 11% increase overall over the next‐best performing tool, GLAM2 (Figures 2c and S4A,B), indicating that it maintains the balance between sensitive detection without over‐prediction. Specifically, SHARK‐capture achieves consistently high performance at both site and residue levels while predicting SLiMs across a range of different lengths between 3 and 10 (*k*_min_ and *k*_max_, respectively, Figure S5). At the site level, GLAM2 offered the highest F1 performance with the highest precision (mean F1 = 0.202, Figures S6 and S7A,E), with SHARK‐capture second‐best (F1 = 0.185) despite recording the highest mean recall (0.193, Figures S6 and S7C). MEME performed poorly with worst‐in‐class performance across all metrics. We further report that SHARK‐capture is able to detect up to 44% of SLiM sites by taking the top 10 consensus *k*‐mer predictions, should sensitivity be of utmost concern. At the residue level, SHARK‐capture recorded the highest average overall performance (mean F1 = 0.129, Figures S6 and S7B), driven by a higher recall (0.111, Figures S6 and S7D) over SLiMFinder, the second‐best performing tool with the highest precision (0.184, compared to 0.176 for SHARK‐capture, Figures S6 and S7F). GLAM2 achieved the highest recall but suffers from poor precision of only 0.06, representing a >60% deterioration over SLiMFinder and SHARK‐capture, since its predictions are usually longer regions that include many non‐ELM‐annotated residues (Figure 2d). MEME was weakest across all performance metrics, with poor detection performance alongside the prediction of longer regions. In parallel, we benchmarked the impact of the post‐processing protocol (SHARK‐capture (extended)). As expected, the extension procedure yielded improved mean site‐ and residue‐level recalls, at the cost of residue‐level precision (Figure 2c,e,f) as indicated by the longer predicted regions (Figure 2d). Surprisingly, we observed a slight increase in site‐level precision due to the merging of multiple overlapping sites, thereby reducing the number of false positive sites. Overall, the post‐processing protocol yielded the highest average F1 performance (Figure 2c) due to improved recall performance.

### 2.3 SHARK‐capture identifies phase separation‐promoting, compositionally biased sites in BuGZ orthologs

**FIGURE 3:**
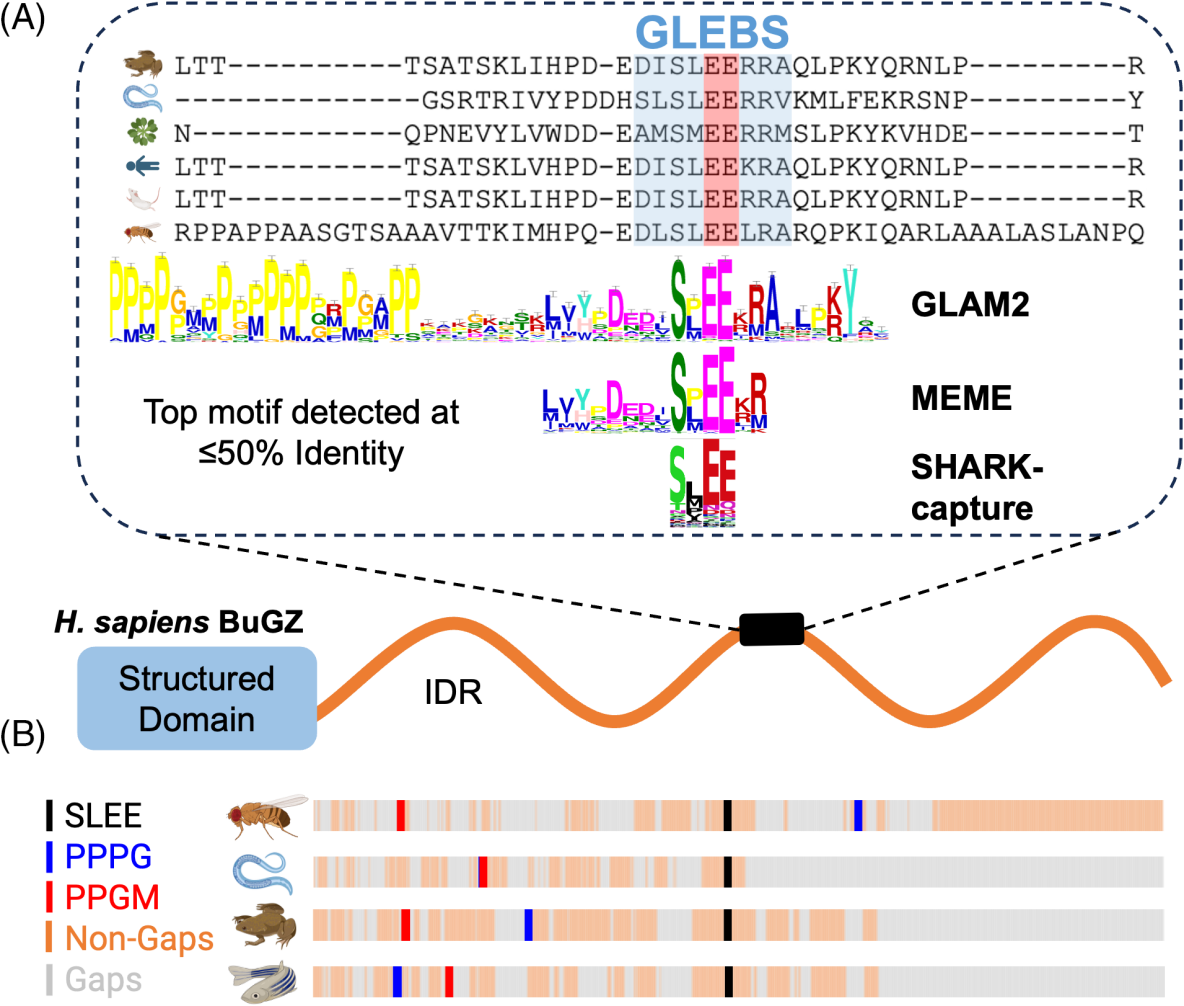
SHARK‐capture identifies a known conserved motif in BuGZ orthologs as well as putative motifs promoting phase separation. (a) *H. sapiens* BuGZ (UniProtID O43670) consists of a structured N‐terminal region and an extended C‐terminal IDR tail, which contains a Bub3 interacting motif with an ultra‐conserved glutamic acid (EE) doublet. (b) Among a set of highly diverged (max. 50% identity) BuGZ orthologous C‐terminal IDRs, SHARK‐capture was capable of detecting the ultra‐conserved glutamic acid doublet, as well as a variety of proline/glycine‐rich consensus *k*‐mers reported to promote phase separation, which are then mapped back onto the sequences of *Drosophila melanogaster*, *Caenorhabditis elegans*, *Xenopus laevis tropicalis*, and *Danio rerio* orthologs via identification of reciprocal best matches (hence not necessarily equivalent to the consensus *k*‐mer)).

As a proof‐of‐concept to assess if SHARK‐capture can detect multiple dispersed motifs, we investigated the ability of SHARK‐capture to identify known motifs in the BuGZ IDR. BuGZ promotes spindle assembly during mitosis, and orthologs are capable of complementing the function when endogenous BuGZ is knocked down in HeLa cells (Chin et al., 2022; Jiang et al., 2014). Interestingly, several BuGZ orthologs tested are also capable of liquid–liquid phase separation (LLPS) in vitro (Chin et al., 2022), indicative of the conservation of LLPS capability. Importantly, it has been shown that phase separation of *X. laevis* BuGZ is required for conferring function (Jiang et al., 2015). Furthermore, BuGZ is known to interact with the mitotic checkpoint protein BUB3 via a conserved GLEBS motif harboring an ultra‐conserved glutamic acid (EE) doublet in its C‐terminal IDR (Jiang et al., 2014; Toledo et al., 2014) (Figure 3a). Accordingly, we investigated if SHARK‐capture could identify the conserved motif among a set of highly diverged (max. 50% identity) orthologous BuGZ C‐terminal IDRs.

SHARK‐capture identified a 4‐amino acid motif, SLEE, as the most conserved, constituting 4 out of the 9 residues of the GLEBS motif (according to the ELM class LIG_GLEBS_BUB3_1, Figure 3a). Although SHARK‐capture only captured part of the GLEBS motif, it nonetheless detected the ultra‐conserved glutamate residues, whereas SLiMFinder was unable to detect any conserved motifs. MEME and GLAM2 were capable of detecting the full GLEBS motif but also included other non‐motif residues, in particular GLAM2, whose predictions in this motif spanned over 100 amino acids.

Interestingly, SHARK‐capture also detected a series of proline and glycine‐rich *k*‐mers in the top 10 predictions, such as PPG, PPPG, and PGLP, conforming to the Pro‐X_0‐4_‐Gly class, which has previously been shown to encode phase separation behavior in disordered proteins (Quiroz & Chilkoti, 2015). Since LLPS is required for BuGZ activity and is conserved amongst orthologs, this highlights the ability of SHARK‐capture to identify functional regions in IDRs. Importantly, these poly‐proline‐glycine‐rich regions are dispersed along the IDR in different orders (Figure 3b), making it difficult for alignment‐based methods to detect all such sites. By contrast, SHARK‐capture was uniquely capable of detecting such regions and mapping them back onto their respective sequences, using the reciprocal matches to find corresponding best matches to the motif (Figure 3b). Altogether, SHARK‐capture is the only tool capable of identifying both the microtubule‐binding GLEBS motif as well as multivalent, compositionally distinct, and phase‐separation‐promoting regions, highlighting its ability to detect multiple conserved functional sites dispersed across the orthologs.

### 2.4 SHARK‐capture identifies a conserved, functional motif among Ded1p orthologs

**FIGURE 4:**
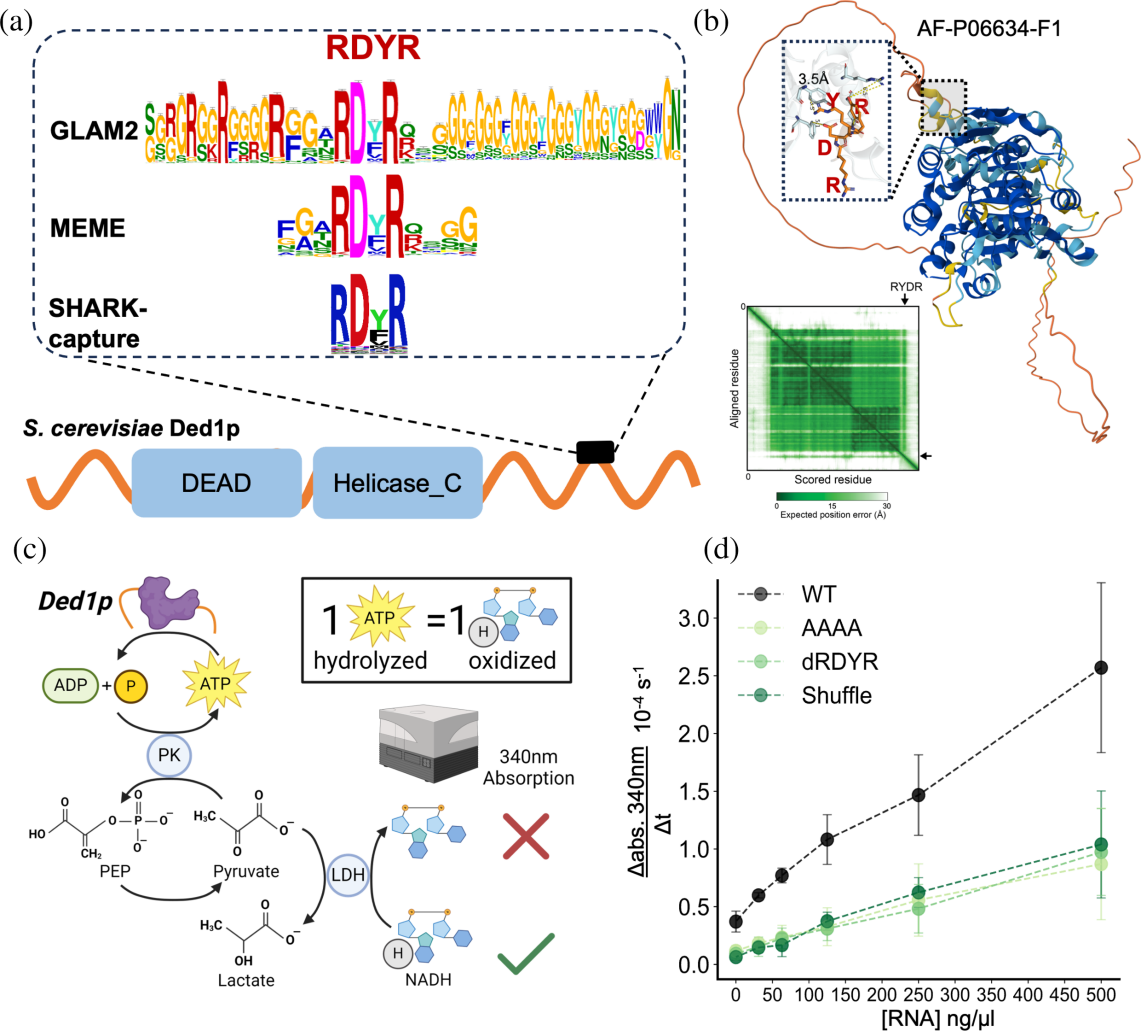
SHARK‐capture identifies a highly conserved motif amongst Ded1p orthologs which regulates ATPase activity. (a) Domain architecture of Ded1p helicase showing the C‐terminal IDR and the RDYR motif detected by SHARK‐capture to be highly conserved. (b) AlphaFold2 structure prediction of Ded1p, showing the RDYR motif and the helix (dashed box) alongside the helicase core (turquoise). AlphaFold predicts close proximity between R557 and Y359, which is in the helicase core (Figure S8a,b). The interaction is further supported by a low position error between the motif and the structured domain (highlighted in PAE matrix) and reruns of AlphaFold2 and 3 with different seeds (Figure S8ac) (c) Overview of the ATPase activity assay, showing the 1:1 stoichiometric relationship between NADH oxidation and ATP hydrolysis. (d) ATPase activity of WT and 3 RDYR motif mutants show a consistent and significant reduction in ATPase activity (measured by rate of change of NADH absorbance) (*n* = 3 for all measurements except [RNA] = 31 ng/nL and WT at [RNA] = 500 ng/nL, where *n* = 2). Raw absorbance traces provided in Figures S9–S11 and Supplementary Datasets.

To further highlight the efficacy of SHARK‐capture, we focused on Ded1p, an ATP‐dependent helicase involved in *S. cerevisiae* translation initiation (Iserman et al., 2020). Among the C‐terminal IDR of Ded1p orthologs, SHARK‐capture identified a 4‐amino‐acid region, RDYR in *S. cerevisiae*, as the top‐ranked prediction (Figure 4a). Interestingly, AlphaFold2 predicts not only a helix around the motif region but also a close proximity (3.5 Å) between residues R557 and Y359 (AlphaFoldDB Model AF‐P06634‐F1, Figures 4b and S8), suggestive of a potential interaction between the RDYR motif and the helicase core. Given its conservation, we hypothesized that the motif (henceforth referred to as the RDYR motif) would be critical for Ded1p helicase activity.

Since Ded1p requires ATP to be active as a helicase (Ebel & Lardy, 1975; Gresser et al., 1982; O'Neal & Boyer, 1984; Pullman et al., 1960), we compared the rate of ATP hydrolysis of wild‐type and variants of Ded1p lacking the RDYR motif. To this end, we used an ATPase assay that is based on the loss of NADH absorbance at 340 nm as a spectrophotometric readout of ATP hydrolysis rate, due to the stoichiometric relationship between ATP regeneration (following hydrolysis) and NADH oxidation (Figure 4c). To investigate the necessity and sufficiency of the RDYR motif in regulating ATPase activity, we generated an alanine scan mutant (AAAA), a deletion mutant (dRDYR) and a mutant where the RDYR motif was translocated to another region in the C‐terminus (shuffle) (see Methods in Section 4 and Table 2). Remarkably, all three mutants showed a consistent reduction in the rate of ATP hydrolysis relative to wild‐type (Figures 4d and S9–S11).

**TABLE 2:** Ded1p variants for ATPase assay.

Variant name	Position	Modification
WT	N/A	None
AAAA	554–557	RDYR to AAAA (alanine scan)
dRDYR/ΔRDYR	554–557	Deletion of RDYR motif
Shuffle	554–557 ➔ 579–580	RDYR motif translocated between G579 and G580

This corroborates a previous report that the large‐scale deletion of the C‐terminal extension of the human homolog DDX3X also inhibited helicase activity (Floor et al., 2016). Coupled with AlphaFold2's helix prediction at the RDYR motif and its propensity to predict possible disorder‐to‐order transitions (Alderson et al., 2023), this suggests a possible mechanism for how the RDYR motif regulates Ded1p ATPase activity. SHARK‐capture was also capable of identifying a highly conserved and well‐studied motif (YVPPHLR) in Ded1p orthologous N‐termini capable of interacting with another RNA helicase eIF4A during translation initiation (Gulay et al., 2020) (Figure S12). Altogether, these results indicate the ability of SHARK‐capture to identify highly conserved and functional regions in IDRs.

### 2.5 Prediction of motifs across the yeast proteome

**FIGURE 5:**
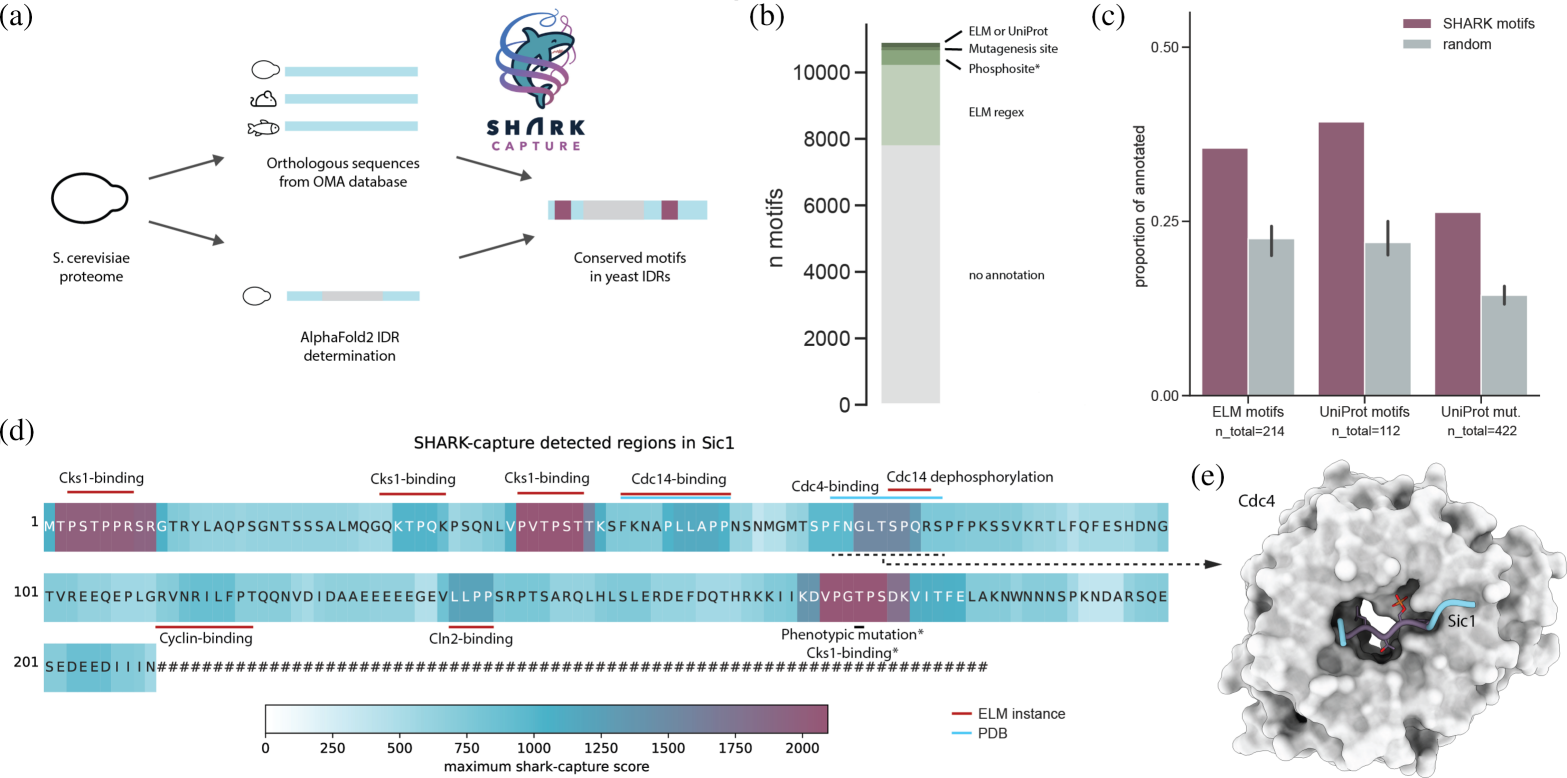
Proteome‐wide prediction of motifs in *S. cerevisiae*. (a) Workflow of the motif prediction for yeast IDRs. (b) Hierarchical annotation of identified motifs, arranged from top to bottom. For a Venn diagram displaying all information, see Figure S14A. Note that phosphosites are not significantly different to randomly sampled *k*‐mers. (c) Matches to ELM motifs, UniProt motifs, and mutagenesis sites (UniProt) are enriched compared to the random control. (d) SHARK‐capture score plot for Sic1. Conserved regions are annotated by ELM or UniProt entries and references (Escoté et al., 2004; Moreno‐Torres et al., 2017). (e) Crystal structure of Sic1 bound to Cdc4 (PDB 3V7D). The structure resolves residues 71–80 of Sic1. Residues 73–77 (colored in purple) correspond to the more conserved *k*‐mer GLTSP.

To test the algorithm on a larger scale dataset and provide a precalculated list of motifs, we ran SHARK‐capture on IDRs of the *Saccharomyces cerevisiae* proteome. For this, we used the OMA database to derive orthologs. SHARK‐capture was run on full sequences, and motifs were returned for disordered regions. Finally, conserved *k*‐mers were assembled and postprocessed, using a cutoff depending on IDR length (see Methods in Section 4) (Figure 5a).

For 2695 SHARK‐capture runs, we calculated 10,889 motifs (11728 sites) in total (Dataset S4). Looking at site‐level matches, 78 and 55 of those are known motifs in ELM and UniProt, respectively. 112 of the motifs contain a residue annotated with a mutagenesis site in UniProt. 71% are unannotated (Figure 5b).

Of the annotated *S. cerevisiae* motifs of ELM and UniProt, we filtered for motifs contained in proteins and IDRs we are considering in our analysis (*n* = 214 and 112). Of those, 36% and 39% are covered by a SHARK‐capture motif, respectively. For annotated mutagenesis sites in UniProt, 26% are covered within SHARK‐capture motifs. When comparing these annotations to randomly sampled *k*‐mers of the same lengths and protein regions, we detect an enrichment for ELM motifs, UniProt motifs, and mutagenesis sites (Figure 5c), while for phosphosites and ELM regex matches, it is close to random (Figure S14B,C).

### 2.6 SHARK‐capture detects functional sites in Sic1 IDP

While many motifs remain unannotated, several well‐studied proteins have multiple known motifs that SHARK‐capture also identifies (Figure S15). An example with the most explained SHARK‐motifs is Sic1 (UniProt P38634) (Figure 5d), a cyclin‐dependent kinase inhibitor in yeast that plays a role in cell cycle regulation. It is a mostly intrinsically disordered protein, with one domain predicted to be more structured at the C‐terminus (Brocca et al., 2009). Of the top five motifs detected by SHARK‐capture, two are known Cks1 binding sites (Kõivomägi et al., 2013). Additionally, one SHARK‐motif overlaps with a Cdc14 dephosphorylation site (Visintin et al., 1998) as well as a Cdc4 binding site solved by x‐ray crystallography (Tang et al., 2012) (PDB 3V7D, Figure 5e); another motif matches with a Cln2 binding site (Bhaduri & Pryciak, 2011; Kõivomägi et al., 2011). In addition, SHARK‐capture detects a motif containing a known mutagenesis site in Threonine 173, which is a known phosphosite of Sic1 (Escoté et al., 2004). Mutation of T173 to Alanine leads to the inability to arrest the cell cycle in response to osmostress, and it has been shown to be an additional Cks1 binding site (Moreno‐Torres et al., 2017). Thereby we can annotate all higher scoring and some lower scoring regions of the Sic1 IDR, showing that SHARK‐capture can identify many different motifs or functional sites.

## 3 DISCUSSION

Increasing efforts to understand the functional elements in IDRs necessitated innovations in the detection of short regions/motifs that are either conserved across evolution or evolved convergently. Accurate computational detection of these regions will benefit experimentalists by providing high‐confidence predictions, which can then be mutated and assayed for functional necessity and sufficiency. Here we report the development of SHARK‐capture, an alignment‐free motif detection tool that incorporates amino acid physicochemical similarity into its core *k*‐mer enumeration‐based algorithm and highlight its efficacy in identifying functional sequence motifs in IDRs.

We performed a systematic benchmark across SLiMs curated in the ELM database to assess the performance of SHARK‐capture compared to a selection of tools and showed SHARK‐capture's superior residue‐level performance at detecting ELMs. We predicted surmize that the higher rate of evolution in IDRs, leading to a greater prevalence of InDels and higher divergence between functional homologs, would render alignment‐based motif detection tools ineffective. This is supported by the performance of the alignment‐based tool GLAM2, which achieves high sensitivity/recall at the cost of greatly lower precision at the residue level, suggesting that it tends to over‐predict the conserved region. This is corroborated by the far longer predictions for the set of BuGZ and Ded1p orthologs; although GLAM2 predicted the region containing the GLEBS and RDYR motifs respectively, it did so by detecting a large region that encompassed the motifs and may not provide the necessary resolution to identify the critical residues that are necessary and sufficient for function. This is particularly relevant if such predictions are to be followed up by experimental validation; the prediction of large regions will not facilitate the efficient identification of functionally critical residues.

Given our belief in the unsuitability of alignment in detecting motifs within IDRs, we also benchmarked against several alignment‐free tools, including MEME which relies on an expectation–maximization strategy. Surprisingly, it achieved consistently poor performance across both site and residue levels. More importantly, however, MEME consistently offers the lowest recall across all tools in the benchmark. It also predicts longer regions that may not be useful for subsequent experimental investigations similar to GLAM2. We also benchmarked the performance of SLiMFinder, a widely adopted alignment‐free enumeration‐based algorithm (Davey et al., 2010). SLiMFinder is an adaptation of long‐standing alignment‐free enumeration algorithms for pattern searches. An early tool, TEIRESIAS, laid the foundational ideas of combinatorial/enumeration‐based search for biological motif discovery (Rigoutsos & Floratos, 1998). However, the initial algorithm only allowed searches for identical patterns/motifs, and whereas later improvements and adaptations including SLiMFinder allowed for non‐identical amino acids to be considered, they nonetheless required the definition of pre‐defined “equivalency groups” (Davey et al., 2010; Exarchos et al., 2011), which applies a rigid blanket assumption that all amino acids within the group are functionally identical. Moreover, TEIRESIAS requires various user‐defined parameters such as the minimum number of sequences containing the motif, the length of the motif as well as the number of non‐conserved (wild‐card) positions, many of which are still required by SLiMFinder. We further note that while the core scanning and convolution steps of SLiMFinder are alignment‐free, it does rely on a BLAST search to define ‘unrelated protein clusters’ (UPCs) to find motifs arising from convergent evolution. This strict requirement for convergent evolution may contribute to the lowered sensitivity in ELM detection across site and residue levels. This may have contributed to the inability of SLiMFinder to offer any predictions for the BuGZ and Ded1p sequences, although multiple UPCs were found via the BLAST search, we are not convinced of the accuracy of such clusters given the ineffectiveness of alignment for IDRs (Chow et al., 2024; Ho et al., 2023; Zarin et al., 2019). Nonetheless, it offered much‐improved performance over MEME in the ELM benchmark and generally predicted shorter motifs of typical ELM lengths, suggestive of the inherent advantages of enumeration‐based algorithms for SLiM detection.

Accordingly, SHARK‐capture aims to improve the enumeration algorithms by reducing the number of user‐input parameters to simplify its use, and to allow flexible assessment of amino acid similarity based on their physicochemical properties instead of requiring equivalency groups. In the former case, the main adjustable parameters for SHARK‐capture are the minimum and maximum lengths of detected motifs *k*_min_ and *k*_max_ (by default set to 3 and 10, respectively). More important is the incorporation of physicochemical information, which allows flexible and nuanced assessment of *k*‐mer similarity. We believe this is an improvement over equivalency groups since the assignment of identically functioning amino acids cannot be made without significant *a priori* understanding of the function and sequence contexts. Contrastingly, the use of a physicochemical distance matrix allows a more balanced consideration of amino acid similarity since it simultaneously considers various physicochemical properties. Another core innovation in our algorithm is the detection of reciprocal best‐matching *k*‐mers, which establishes bilateral correspondence, which could be indicative of conservation. Altogether, these improvements are reflected in the ability of SHARK‐capture to detect SLiM sites with the highest recall. Simultaneously, the removal of non‐reciprocal matches acts as a noise filter for regions without strong support for conservation, at least according to physicochemical properties. This should leave only highly conserved regions across a set of sequences which is reflected in the high precision at the residue level, indicating that unlike MEME and GLAM2, they do not rely on the prediction of longer regions to guarantee a higher recall. Ultimately, improvements to both precision and recall result in SHARK‐capture offering strong overall performance (assessed by F1‐score) at identifying SLiMs across all classes (site level) and accurately identifying the SLiM within the sequences with high resolution (residue level).

We also highlighted potential scenarios where SHARK‐capture may be used to detect functional motifs, which could then be verified experimentally. The set of BuGZ sequences served as an initial proof‐of‐concept to assess the ability of SHARK‐capture to identify the known conserved GLEBS motif within the set of orthologs. The ability of SHARK‐capture to identify the SLEE region suggests that while it was unable to achieve full recall of the GLEBS motif, its detection is of high precision, contrary to GLAM2 and MEME, where non‐motif residues were also predicted. This highlights a recall‐precision tradeoff to consider when choosing motif detection tools and also hints at the benefit of combining the predictions of multiple tools. On the other hand, SHARK‐capture is unique in its ability to detect the Pro‐X_0‐4_‐Gly and P/G‐rich regions shown to promote LLPS in vitro, which is required and conserved for BuGZ function. Importantly, these sites are dispersed in different orders throughout the sequence since they represent a distinct functional class. They are therefore difficult to detect by alignment, although we were nonetheless surprised by the inability of MEME and SLiMFinder to retrieve such regions at all. With increasing evidence of the role of multivalent interactions in driving IDR function (particularly in the formation of biomolecular condensates through LLPS) (Banani et al., 2017; Fenton et al., 2023; Fung et al., 2018; Jo & Jung, 2019), SHARK‐capture could potentially meet the demand for tools that facilitate the systematic discovery of short motifs interspersed throughout the sequence. It is the only tool among those tested in this investigation to be able to identify sets of conserved local low‐complexity regions harboring biased amino acid compositions which may drive multivalent IDR functions including LLPS and the formation of biomolecular condensates.

Analysis of the RDYR motif in Ded1p further indicates the efficacy of SHARK‐capture in detecting functional motifs in IDRs. This is evidenced by the strong reduction in ATPase activity following the mutation of only 4 amino acids. While the ATP hydrolysis rate is not a direct readout of Ded1p helicase activity, we believe that a reduction in ATP hydrolysis rate would likely impact its helicase function. This is substantiated by the increase in ATP turnover when RNA concentration is increased, suggesting that RNA concentration is rate‐limiting (at least *in vitro*). Interestingly, the RDYR motif appeared to be positionally constrained since the shuffle mutant also showed reduced activity, potentially due to steric constraints in its interaction with the helicase core. This is also supported by the presence of a predicted helix around the motif region, perhaps indicative of a possible disorder‐to‐order transition upon interaction with the helicase core; further experiments are required to completely elucidate the underlying mechanism of its regulatory effect. Nonetheless, the detection of a short motif and supporting evidence of its functional impact highlights the improved resolution and precision SHARK‐capture can provide. Whereas Floor *et al.* had already shown an impact on helicase activity following the deletion of the C‐terminal extension (Floor et al., 2016), the large‐scale deletion did not yield the necessary resolution to identify specific residues or motifs critical to function, while SHARK‐capture detected the functional motif with greater precision. Consistent with the performance on the BuGZ IDRs, SLiMFinder failed to detect any region, GLAM2 basically detected the entire C‐terminus, and MEME also identified a longer region. We do note, however, that in this case, the longer region is not necessarily indicative of over‐prediction since it is also possible that the mutation of the longer region further reduces ATPase activity. Nonetheless, the ability of SHARK‐capture to precisely delineate a short region capable of significantly modulating Ded1p activity again highlights the advantage of SHARK‐capture over existing tools in providing confident and precise predictions of functional motifs in IDRs.

We want to note that the exact boundaries of SLiMs are often unknown and may be even organism‐ and sequence‐specific, further complicating both experimental design and precise benchmarking. It has been shown that other sites outside the strict SLiM definition contribute to binding affinities, for example, in the case of the TRFH domain binding motif of TIN2 (Chen et al., 2008). In addition, variants of the same domain can influence the binding affinities to a motif, as seen in, for example, SH3 domains (Rouka et al., 2015). Several examples are known when the removal of disordered regions that do not physically contact the binding partner decreases the binding affinity (Zor et al., 2002; Selenko et al., 2003). Such complexes, when structural disorder is maintained in the bound state, are termed fuzzy complexes in a seminal paper by Fuxreiter and Tompa (Tompa & Fuxreiter, 2008). While more systematic testing and reporting would be necessary on the impact of motif flanking or even distant regions on the binding affinity of SLIMs. We hope that SHARK‐capture facilitates the discovery of conserved flanking regions that do not directly contact the binding partner but contribute to binding affinity.

To facilitate systematic discoveries of motifs, we calculated motifs for yeast IDRs and provided them as a resource (Dataset 4). Our results show that we can capture many previously known motifs and mutagenesis sites. However, due to limited information on many IDRs, many SHARK‐capture motifs remain unexplained. Focusing on the well‐studied protein Sic1, we show that SHARK‐capture returns sensible motifs, even for lower‐scoring sites. This is in contrast to standard benchmark approaches in which only the top motif is considered for evaluation.

For this proteome‐wide analysis, we adopted the optional post‐processing step, which extends matched regions to include suitable matches within a score threshold. This results in improved recall with a slight cost in precision, as indicated by the performance on the ELM benchmark, which may be beneficial in detecting motifs at a proteome‐wide level. Of course, users can vary the inclusion threshold to further bias towards the extension of longer regions to further increase motif recall at the cost of precision, but we note that the extension procedure was developed to complement the weighting strategy (see Materials and Methods in Section 4: SHARK‐capture algorithm, step 7) in identifying longer regions that are still thoroughly highly conserved. Accordingly, the inclusion threshold (90% of the top‐ranked SHARK‐capture score) is strict. As an example, the residues flanking the GLEBS motif were not included even with the extension protocol because these surrounding residues are highly degenerate, as indicated in the ELM database (represented as [EN][FYLW][NSQ].EE[ILMVF][^P][LIVMFA]) and in other GLEBS motif alignments (Jiang et al., 2014; Toledo et al., 2014). Here, SHARK‐capture's predicted SLEE region lies in concordance with the most conserved region within the GLEBS motif, as shown by Toledo *et al.* (Toledo et al., 2014). We therefore believe that if relatively short regions are still consistently predicted even after the extension procedure, this may be suggestive of the markedly high conservation of these small regions (even within their sequence context) with interesting functional implications.

Despite increased runtime and memory requirements relative to the other tools benchmarked in this study (Figure S16 and Table S1), SHARK‐capture can still be run locally for datasets with up to a few hundred sequences. For larger datasets, we provide scripts for increased parallelization for use in high‐performance computing clusters. Therefore, the performance benefits afforded by SHARK‐capture, which have been highlighted in systematic benchmarks as well as in specific use cases, may provide a useful, simple‐to‐use motif detection tool for the detection of conserved, functional motifs in IDRs.

Ultimately, SHARK‐capture aims to facilitate the discovery of the sequence determinants that underlie the plethora of functions of IDRs and contribute to understanding sequence–function relationships in the disordered protein universe.

## 4 MATERIALS AND METHODS

### 4.1 SHARK‐capture algorithm

The core of the SHARK algorithm is described in Chow et al. (2024), and the subsequent adaptations for SHARK‐capture are detailed in the results. For convenience, we summarize the steps of the algorithm that are relevant to SHARK‐capture (steps 1–5) here, but for full details, please refer to Chow et al.:

1. Each sequence is decomposed into overlapping subsequences of length *k* (*k*‐mers). Each sequence is then represented by a vector encoding the frequency of each *k*‐mer.
2. The physicochemical similarity score between each amino acid is calculated using Grantham's Distance matrix (*G*) (Grantham, 1974), where *D′* is Grantham's distance between the pair of amino acids.
3. The similarity between two *k*‐mers (*i* and *j*) is calculated from the average of their index‐wise physicochemical similarity score (*D*).
4. All unique *k*‐mers are compared between two sequences to form a similarity matrix *M*.
5. For each *k*‐mer in *M*, only the most similar *k*‐mer in the other sequence is selected; the rest are filtered out. In cases where there are multiple best matches (multiple *k*‐mers with same similarity), the best matching *k*‐mer is chosen according to the lowest LD value:
6. Reciprocal best matches are thus defined as *k*‐mer pairs where *k*‐mer *i* is most similar to *k*‐mer *j* and *k*‐mer *j* is also most similar to *k*‐mer *i*. In terms of the matrix, this represents values where the row and column best matches intersect.
7. However, comparison of raw scores is only relevant within a particular *k*‐mer length, since longer *k*‐mers generally score lower. We reason that this is, in no small part, due to the greater number of *k*‐mers it could match to and vice versa, which reduces the likelihood of a reciprocal best match (the number of unique *k*‐mers theoretically scales as 20^*k*^, but this maximum would never be reached at higher *k*'s). Accordingly, we employ a weighting factor $f\left(S_{k}\right)=\sqrt[{2}]{\left|S_{k}\right|}$ that scales with the actual number of unique *k*‐mers, which we call the “search space” *S*_*k*_, for a given *k*‐mer length within the set of sequences. Multiplication of the raw score by the weighting factor allows a fairer comparison of *k*‐mer conservation across *k*‐mer lengths and allows the most conserved, top‐scoring consensus *k*‐mers, that is, potential motifs, to be identified.
8. Finally, each *k*‐mer can then be mapped back to a sequence by identifying and locating its most frequent reciprocal match within the sequence (if any).
9. For the all‐vs‐all comparison between sequence pairs in the input set, only unique pairs are considered since comparisons are symmetrical. For a given input set of *n* sequences, this results in *n*(*n* + 1)/2 comparisons (including self‐comparisons). Accordingly, the time complexity of the algorithm is *O*(*n* ^2^).

$$\mathrm{LD}\left(q_{i},t_{j}\right)=\left(\frac{\sqrt{2\left({q_{i}}^{2}+{t_{j}}^{2}\right)}}{q_{i}+t_{j}}\right),$$

as described in Chow et al. (2024), where *q*_*i*_ and *t*_*j*_ are the frequencies of *i* and *j*, respectively.

### 4.2 Post‐processing of SHARK‐capture outputs to extend motifs

To increase motif detection recall, a post‐processing (extension) protocol was developed to extend the top‐ranked SHARK‐capture matches (with SHARK‐capture score *C*) to include lower‐ranked regions if (1) they overlap with the top‐ranked region and (2) their SHARK‐capture score is within 10% of the top‐ranked SHARK‐capture score (i.e., >0.9*C*). This process is sequence‐specific and yields longer motifs that may exceed *k*_max_.

This protocol is applied both to the ELM benchmark and to the proteome‐wide prediction of *S. cerevisiae* IDR motifs. For the ELM benchmark, only the first, top‐ranked consensus k‐mer (i.e., the same consensus k‐mer reported in the SHARK‐capture benchmark), and only regions (k‐mers) mapped by the top 10 SHARK‐capture consensus k‐mers (i.e., first 10 consensus k‐mers sorted by descending SHARK‐capture score) are considered and reported by the post‐processing protocol.

As with all other SHARK‐capture runs reported in this manuscript, the list of the top 10 consensus *k*‐mer instances is reported in Dataset 1 in each ELM class.

### 4.3 Benchmark on short linear motifs (SLiMs) from the eukaryotic linear motif (ELM) database

All 327 ELM classes, 4029 SLiMs (referred to as ELM instances in the ELM database), and corresponding sequence FASTA files were downloaded as of 30th June 2023 (Kumar et al., 2022). Seven SLiMs containing undefined or non‐canonical amino acids were removed. Since the benchmark involves finding true motifs and assumes every sequence contains at least one true motif (i.e., a SLiM), only ELM‐annotated true positive SLiMs were accepted. We further filtered out 11 SLiMs where the SLiM does not match the ELM regex, leaving 3783 SLiMs across 312 ELM classes. According to ELM, an ELM class consists of SLiMs sharing the same function and conforming to the same ELM‐designated regular expression representation of the SLiM.

The SLiMs were then grouped by ELM class, and a FASTA file for each ELM class was created if there were at least 3 unique sequences within the ELM class (we consider motif detection with 2 sequences too unreliable). Sequences are also required to have a minimum length of 10 residues; accordingly, 1 SLiM was filtered out (ELMI004007 with sequence TKPR). Correspondingly, each FASTA file contains full‐length unique sequences (unique sequence accessions); choosing unique sequences prevents any bias since there may be multiple SLiMs of the same ELM class in each sequence. This filtering results in a final dataset of 3679 SLiMs across 252 ELM classes.

Besides SHARK‐capture, the performances of popular motif detection tools MEME (v5.5.3), GLAM2 (v5.5.3) and SLiMFinder (v.5.4.0) were also assessed on this benchmark dataset. All tools were run with default parameters except for SLiMFinder (with BLAST version 2.13.0) where disorder masking was turned off (dismask = F) so that all SLiMs could be found, and interactivity suppressed (*i* = −1) for automated processing.

Tool performance on the benchmark dataset is assessed for each ELM class separately, at site and residue levels similar to Prytuliak et al. (2017). A site is defined as a continuous stretch of residues, either corresponding to a SLiM (annotated positive) or the predicted motif output of a tool (predicted positive). Site‐level performance is assessed by precision‐recall as defined in Table 3.

**TABLE 3:** Site‐level assessment of motif detection performance.

SITE	Tool predicted	Not predicted by tool
ELM‐annotated	True positive (TP): sites predicted by the tool share at least 1 common residue with ELM annotation	False negative (FN): sites annotated in ELM not detected by tool
Not annotated in ELM	False positive (FP): sites where none of the residues predicted by the tool share a common residue with ELM‐annotated sites	NOT CONSIDERED. At the site level, we do not consider true negatives in the calculation of precision and recall since the focus is on detection of annotated sites.

Recall (otherwise known as sensitivity) is calculated as TP/(FP + FN) and precision as TP/(TP + FP). Across all ELM classes, we also report the mean recall and precision over the entire dataset, treating each ELM class with equal weight. We note that because we use unique sequences which may harbor multiple SLiMs of the same type, we allow a predicted site to overlap with multiple annotated sites and count them each as an instance of a true positive. Where no residues are predicted, recall and precision = 0. Whereas this can advantage tools that predict long motif stretches, for example, MEME/GLAM2, they will correspondingly be penalized at the residue level metrics, described below.

Residue level performance is assessed by the metrics of precision‐recall and receiver operating characteristics as defined by the following. Since some SLiMs may overlap, we consider only unique residues when assessing performance as defined in Table 4. Where no residues are predicted, recall and precision = 0. All reported site‐level metrics represented the unweighted mean across all 252 ELM classes. For example, the F1 for site‐level performance is the unweighted mean of the F1 performance across all 252 classes. All reported residue‐level metrics are calculated after aggregation over all sequences within a class (i.e., TP, TN, FP, and FN are summed over all sequences in the class), and the performance is the unweighted mean across all 252 classes. This prevents classes with more SLiMs/sequences from biasing the mean.

**TABLE 4:** Residue‐level assessment of motif detection performance.

RESIDUE	Tool predicted	Not predicted by tool
ELM‐annotated	True positive (TP): unique residues predicted by tool that belong to an annotated site	False negative (FN): unique residues belonging to an annotated site not predicted by the tool
Not considered ELM	False positive (FP): unique residues predicted by tool that are not ELM annotated SLiMs	True negative (TN): unique residues not predicted by the tool that are not in ELM‐annotated regions

Importantly, we apply a highly stringent criterion for SLiM detection, only choosing the top‐ranked predicted SLiM; this is because there is only one shared SLiM for each ELM class. We consider this the most practical way of benchmarking, since we believe this is how experimentalists would use the tool. Where there are multiple top‐ranked SLiMs, we take the first instance of the prediction without any selection. In cases where there are multiple SLiMs within the same sequence, it depends on the respective tool (using their default parameters) as to whether instances of multiple SLiMs of the same ELM class are detected.

### 4.4 Performance of SHARK‐capture using multiple matches on the ELM benchmark

The matches of the top 10 SHARK‐capture‐detected consensus *k*‐mers were combined to assess the potential benefits in SLiM detection by considering multiple SHARK‐capture matches. As with all other SHARK‐capture runs reported in this manuscript, the list of the top 10 consensus *k*‐mer instances and all matches are reported in Dataset S1 for each ELM class.

### 4.5 BuGZ orthologous C‐terminus sequences

The multiple sequence alignment of eukaryotic orthologs (belonging to the eukaryotic orthologous group) of *H. sapiens* BuGZ/ZNF207 (UniProt ID PO43670) was extracted using the eggNOG orthology database (v5.0) and manually curated (eggNOG uses the longest isoform PO43670‐4) (Huerta‐Cepas et al., 2019). From the alignment, the microtubule‐binding domain, manually defined as *H. sapiens* BuGZ residues 1–93, was removed, leaving the C‐terminus. The resulting sequences were filtered for max. 50% identity using CD‐HIT v4.6 (L et al., 2012), and sequences containing non‐canonical amino acids were removed to give a final set of 113 orthologous BuGZ C‐terminal sequences.

### 4.6 Ded1p orthologous N‐ and C‐terminus sequences

Eukaryotic orthologs belonging to the eukaryotic orthologous group of *S. cerevisiae* Ded1p (UniProt ID P06634) were extracted using the eggNOG orthology database (v5.0) and manually curated. Using MAFFT (v7.453) (Katoh et al., 2002) to generate a multiple sequence alignment (MSA) with the orthologs, the MSA was manually curated after the removal of redundant, highly similar (>95% identity) sequences with CD‐HIT. Following this, the N‐ and C‐terminal IDRs were obtained according to the helicase core domain boundaries of the *S. cerevisiae* sequence, defined as positions 99–535 according to alignment with the human ortholog DDX3X for which a crystal structure is available (PDB 2I4I, 4PXA, 5E7I), and further manually cleaned (including the removal of sequences <10 amino acids long and replacement of the yeast paralog 4932.YPL119C with human ortholog DDX3X (9606.ENSP00000382840)). This gave a final set of 268 sequences for each terminus.

### 4.7 Prediction of *S. cerevisiae* motifs

To predict motifs for the *S. cerevisiae* proteome, we used the OMA orthology database (Altenhoff et al., 2021) to collect orthologs. Of the 6060 proteins in the *S. cerevisiae* reference proteome (UniProt release 2024_03), 5393 corresponded to an OMA group. For each group, redundant (highly similar) sequences were removed using CD‐HIT (v4.8.1) (L et al., 2012) with an identity threshold of 80%. In cases where fewer than 5 sequences remained, a more lenient threshold of 90% was used. A final filtering step removed groups with fewer than 5 sequences (even at 90% identity) to give a final set of 4941 *S. cerevisiae* orthologous groups.

SHARK‐capture was run on these sequence groups with default settings (*k*_min_ = 3, *k*_max_ = 10). Conserved *k*‐mers were mapped back to the *S. cerevisiae* sequence. If a *k*‐mer had multiple equal similar matches to the sequence, all positions were returned. We only considered motifs that mapped to an IDR assigned using AlphaFold (v4) models (2901/4941 yeast proteins with IDR) and were more than 2 residues away from a structured region. We then compiled the outputs into one dataset. For each protein, we allowed two motifs per 100 residues of IDR. If multiple equal scoring positions of the same *k*‐mer existed, all were returned. OMA sequences were aligned back to UniProt and corresponding AlphaFold model sequences. For the global output, *k*_min_ of 4 was used to avoid too short motifs. Motifs were extended as described below.

For a random control, we sampled a *k*‐mer of the same length from the same protein sequence as the original *k*‐mer. This random sampling process is repeated five times for each k‐mer.

### 4.8 IDR determination from AlphaFold predictions

To identify IDRs, we used a sliding window of 15 residues to average pLDDT values. Residues with a pLDDT ≤65 were considered disordered. Similar to Tesei et al. (2024), we then reassigned small regions of order or disorder, if they were ≤10 residues and flanked by order or disorder (first reassigned ordered). We considered a minimal IDR length of 30 residues.

### 4.9 Runtime and memory benchmarks

A subset of sequences (of sizes 5, 10, 30, 50, 100, and 200) was randomly sampled from the set of Ded1p orthologous C‐terminus sequences. For each set of sequences, the runtime and peak memory usage for each motif detection tool were recorded. All tested tools were run on a Quad‐core 2.4 GHz Intel machine with 16GB of RAM. Since SHARK‐capture offers parallel processing, all 4 cores were utilized using the (−n_processes 4) parameter. Other tools were run using default settings. The results are shown in Figure S16 and Supplementary Table S1. Several SLiMFinder runs were prematurely aborted due to an insufficient number of unrelated protein clusters (UPCs) found, which are required for subsequent steps.

### 4.10 Ded1p mutant variants

Ded1p mutants are expressed as described in Supp. Methods (Figure S12). Besides WT, 3 mutants of the RDYR motif (Table 2) are also expressed and purified for assessment of ATPase activity (Figure S13).

### 4.11 ATPase activity assay

Concentrations of all Ded1p variants were prediluted in sample buffer (50 mM Tris/HCl pH 8.0, 1 M KCl, 2 mM EDTA, 1 mM DTT) and normalized to WT concentration. 3 μL of ~6.7 μM sample (slight variation depending on measured WT concentration per replicate) was then added to 15 μL of 2× assay buffer (100 mM HEPES/KOH at pH 7.45, 20 mM MgCl_2_, 2 mM DTT) in a non‐binding 384‐well plate (Greiner Bio‐One). PolyA RNA (Merck, Darmstadt, Germany) was serially diluted (twofold) in water, with 2 μL added to the reaction mix. 0.23 μL of lactate dehydrogenase–pyruvate kinase enzyme mix (Merck, Darmstadt, Germany) was added to each sample to give a volume of 20 μL. Here, the final concentration of protein is ~1 μM in assay buffer (50 mM HEPES/KOH pH 7.45, 10 mM MgCl_2_, 1 mM DTT, 2.7 mM PEP, and 0.27 mM NADH). 100 mM ATP stock was 4× diluted in 1× assay buffer (50 mM HEPES/KOH at pH 7.45, 150 mM KCl, 10 mM MgCl_2_, and 1 mM DTT), and 5 μL was added to each well. NADH absorbance at 340 nm was then measured using a Tecan Spark 20 M microplate reader at 25°C with shaking.

For each sample, generally allowing several minutes for the initial equilibration of ATP with the rest of the reaction mixture, the steady‐state rate of change in NADH absorbance was measured within the linear regime and calculated as $\frac{∆\text{NADH absorbance}\;\left(340\mathrm{nm}\right)}{∆\text{time}}$ over a given time interval (>200 s). For higher RNA concentrations where the rate of reaction is very high, the initial rate of reaction is taken (i.e., from *t* = 0 s) only if the decrease in NADH absorbance is linear, else it is not reported as it cannot be accurately measured.

## AUTHOR CONTRIBUTIONS

**Chi Fung Willis Chow:** Conceptualization; methodology; data curation; investigation; validation; formal analysis; visualization; writing – original draft; writing – review and editing. **Swantje Lenz:** Methodology; formal analysis; investigation; visualization; writing – review and editing; data curation; validation. **Maxim Scheremetjew:** Software; writing – review and editing. **Soumyadeep Ghosh:** Software. **Doris Richter:** Investigation; writing – review and editing. **Ceciel Jegers:** Investigation; resources; writing – review and editing. **Alexander von Appen:** Supervision; writing – review and editing. **Simon Alberti:** Supervision; writing – review and editing; funding acquisition. **Agnes Toth‐Petroczy:** Writing – review and editing; conceptualization; investigation; funding acquisition; visualization; project administration; supervision.

## CONFLICT OF INTEREST STATEMENT

S. A. is an advisor on the scientific advisory board of Dewpoint Therapeutics.

## Supporting information


**Data S1.** Supporting Information.

## Data Availability

The code base and readme files can be found at https://doi.org/10.5281/zenodo.14144665, and the information on the benchmarking datasets and results are available as Supplementary Datasets S1–4 at https://doi.org/10.17617/3.TGOQYO. SHARK‐capture is available as a Python package (https://pypi.org/project/bio-shark/) and on git (https://git.mpi-cbg.de/tothpetroczylab/shark) where we provide bash scripts for parallelization on HPC and a colab notebook https://colab.research.google.com/drive/1l_hNYr8OzfHAv9u4HOytB4LNLVbIIL2z?usp=sharing.
