# Supplementary material for: SHARK‐capture identifies functional motifs in intrinsically disordered protein regions
Source: Protein Sci. 2025 Mar 18;34(4):e70091. doi: 10.1002/pro.70091 (PMC11917139; doi:10.1002/pro.70091)
Supplement: Supplementary file 1 — Data S1. Supporting Information. [file PRO-34-e70091-s001.pdf]

Supplementary Information

for

SHARK-capture identifies functional motifs in intrinsically disordered protein regions

Chi Fung Willis Chow[1,2,3#], Swantje Lenz[1,2#], Maxim Scheremetjew[1,2], Soumyadeep Ghosh[1,2], Doris Richter[1], Ceciel Jegers[3,4], Alexander von Appen[1,2], Simon Alberti[3,4], Agnes Toth-Petroczy[1,2,3*]

1. Max Planck Institute of Molecular Cell Biology and Genetics, Pfotenhauerstrasse 108, 01307 Dresden, Germany
2. Center for Systems Biology Dresden, Pfotenhauerstrasse 108, 01307 Dresden, Germany
3. Cluster of Excellence Physics of Life, TU Dresden, 01062 Dresden, Germany
4. Biotechnology Center (BIOTEC), Center for Molecular and Cellular Bioengineering, Technische Universität Dresden, Tatzberg 47/49, 01307 Dresden, Germany

#equal contribution

*corresponding author, toth-petroczy@mpi-cbg.de

Supplementary Methods

Cloning and mutagenesis

Wild-type pUC57-DED1-Kanamycin plasmids were kindly provided by Ceciel Jegers (Alberti Lab, BIOTEC, TU Dresden). In accordance with the protocol from Jegers et al. [1] and Iserman et al. [2], mutant sequences were codon optimized for Sf9 expression (IDT codon optimization tool), ordered as gBlocks (IDR, Leuven, Belgium) and restriction digested with SapI. pUC57-DED1-Kanamycin plasmids (Genscript, Rijswijk, Netherlands) were also SapI-restricted digested and gel extracted (QIAprep Gel Extraction kit, QIAGEN, Hilden, Germany). gBlocks were subsequently ligated with pUC57 vectors using quick ligation (NEB, Frankfurt am Main, Germany). Proper cloning was verified with colony PCR and sequencing using standard primers. For virus production, the DED1 gene (wild-type and variants) was subcloned into pOCC120 shuttle vectors using AscI and NotI restriction sites (NEB, Frankfurt am Main, Germany) [3] and quick ligation, followed by verification with sequencing.

Bacterial transformation and plasmid extraction

Following the protocols from Jegers et al. [1] and Iserman et al. [2], *E. coli DH5α* cells were thawed on ice in 50 µL ligation volume (KCM buffer 0.1 M KCL, 0.03 M $CaCl_2$, 0.05 M $MgCl_2$ + 5 µL plasmid). Following 1 minute heat shock at 42 °C, cells were placed on ice for 5 min, followed by 1 hour recovery at 37°C with shaking in LB medium (1% peptone, 0.5% yeast extract, 1% NaCl). Bacteria were then streaked on kanamycin (pUC57) or ampicillin (pOCC120) plates and incubated overnight at 37°C. Clones were picked and inoculated in 5 mL LB with antibiotics and left overnight at 37°C and shaking. Plasmids were extracted using QIAprep Spin Miniprep Kit (QIAGEN, Hilden, Germany).

Protein purification

Recombinant MBP-3C-DED1-3C-monoEGFP (wild-type and variants) were expressed for 48 hours in *T.ni* insect cells using a baculovirus expression system [3]. Subsequent steps largely followed the protocol of Jegers et al [1] and Iserman et al. [2]. Cells were lysed at 15000 PSI on ice with an LM20 microfluidizer (Microfluidics, Westwood, USA) in sample buffer (50 mM Tris/HCl pH 8.0, 1 M KCl, 2 mM EDTA, 1 mM DTT), with Benzonase (MPI-CBG) and protease inhibitor (Merck, Darmstadt, Germany) added. Protein-containing lysate was then collected after 1 hour ultracentrifugation ~32000*g* at 6 °C. The supernatant was then incubated with amylose resin (New England Biolabs) for 1 hour at 4 °C. Following 3 column-volume washes, MBP-tagged protein was eluted with sample buffer + 20mM maltose. MBP was then cleaved off overnight with Prescission (3C, MPI-CBG) protease at 4°C. Samples were then concentrated with 30K Vivaspin centrifugal filters (Vivaproducts, Littleton, USA) then applied to size exclusion chromatography in a Superdex 200 pg 10/30 (Cytiva, Marlborough, USA) using an ÄKTA pure 25 (GE Life Sciences, Germany) at room temperature. GFP-containing fractions were pooled (absorbance at 488 nm), further concentrated, and stored at -80°C following flash freezing. Samples were later also analyzed by SDS-PAGE gel (Figure S13). Before subsequent experiments, protein concentration was determined by measuring absorbance at 488 nm using an NP-80 UV/VIS nano spectrophotometer (Implen, Munich, Germany).

Performance of SHARK-capture using multiple matches on the ELM benchmark

The matches of the top 10 SHARK-capture-detected consensus k-mers were combined to assess the potential benefits in SLiM detection by considering multiple SHARK-capture matches. As with all other SHARK-capture runs reported in this manuscript, the list of the top 10 consensus k-mer instances are reported in sharkcapture_top10_consensus_kmers.csv in each ELM class, and all matches are reported in sharkcapture_top10_occurrences.tsv in the benchmark output folder. The number of SLiM sites detected (i.e. at least 1 residue overlap between predicted and real site) across all 252 ELM classes is reported in the text.

Supplementary Figures

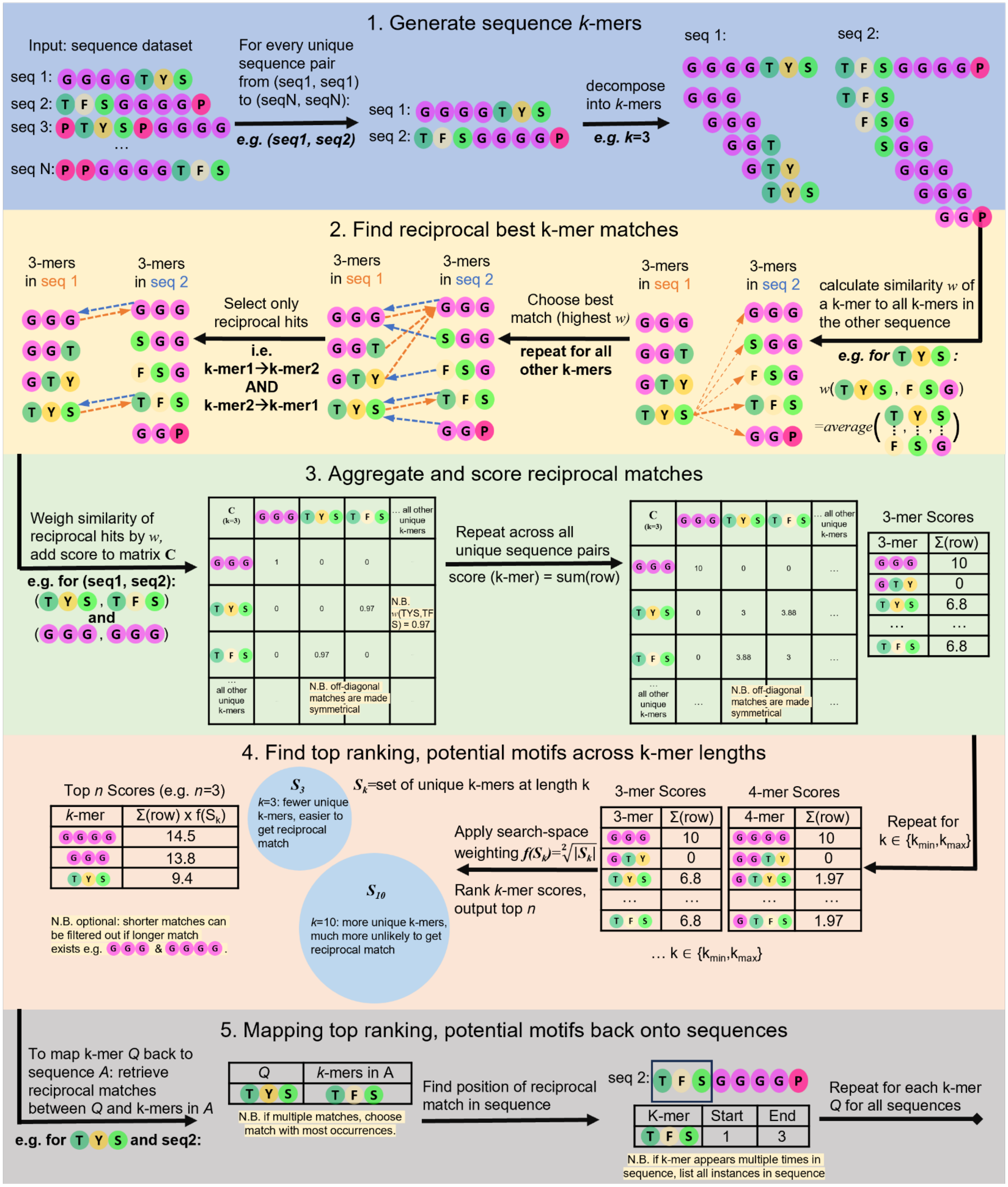

Figure S1. SHARK-capture steps in detail. 5 main steps of the SHARK-capture algorithm are shown. Step 1 is identical to other alignment-free word-based algorithms. The core innovation is in step 2 where reciprocally most-similar (likely conserved) *k*-mers are identified. These reciprocal matches are aggregated and scored to identify the most conserved consensus *k*-mers, which are then compared and ranked across *k*-mer lengths (step 4). Finally, these consensus *k*-mers are mapped back onto each sequence (step 5).

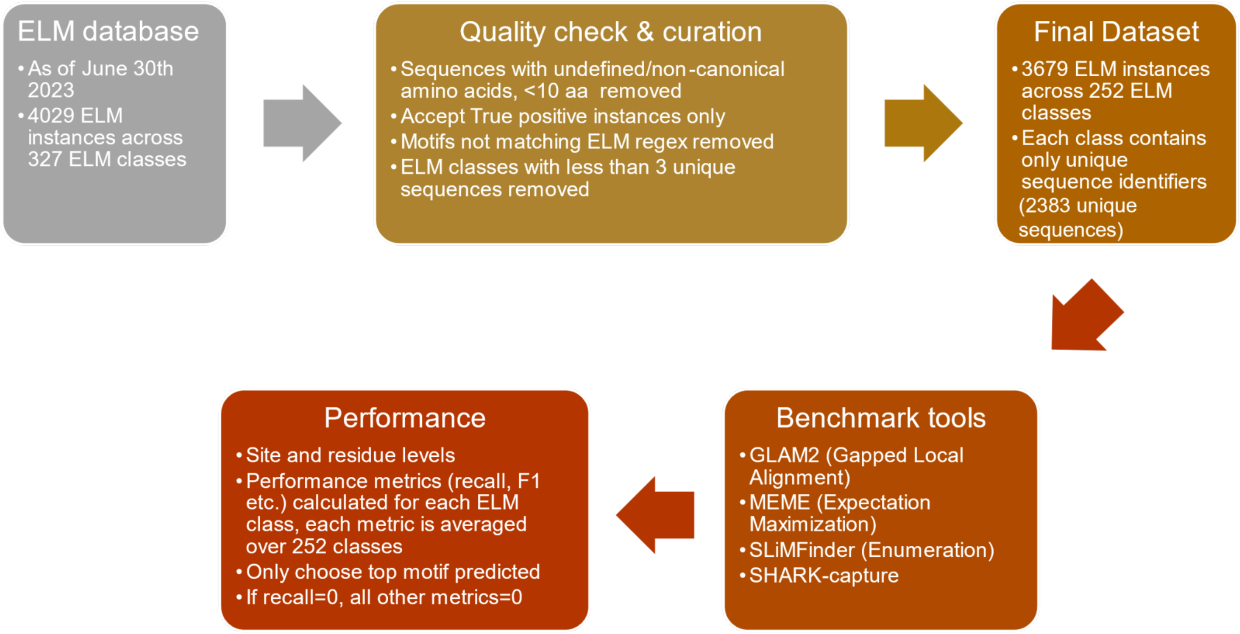

Figure S2. Systematic benchmarking of SLiM detection performance in detail. The ELM benchmark dataset was curated following several quality filtering steps, and to allow for a sufficient number (3) of unique UniProt sequences. The result is 3679 SLiMs across 252 ELM classes, and the performance of 4 tools is compared.

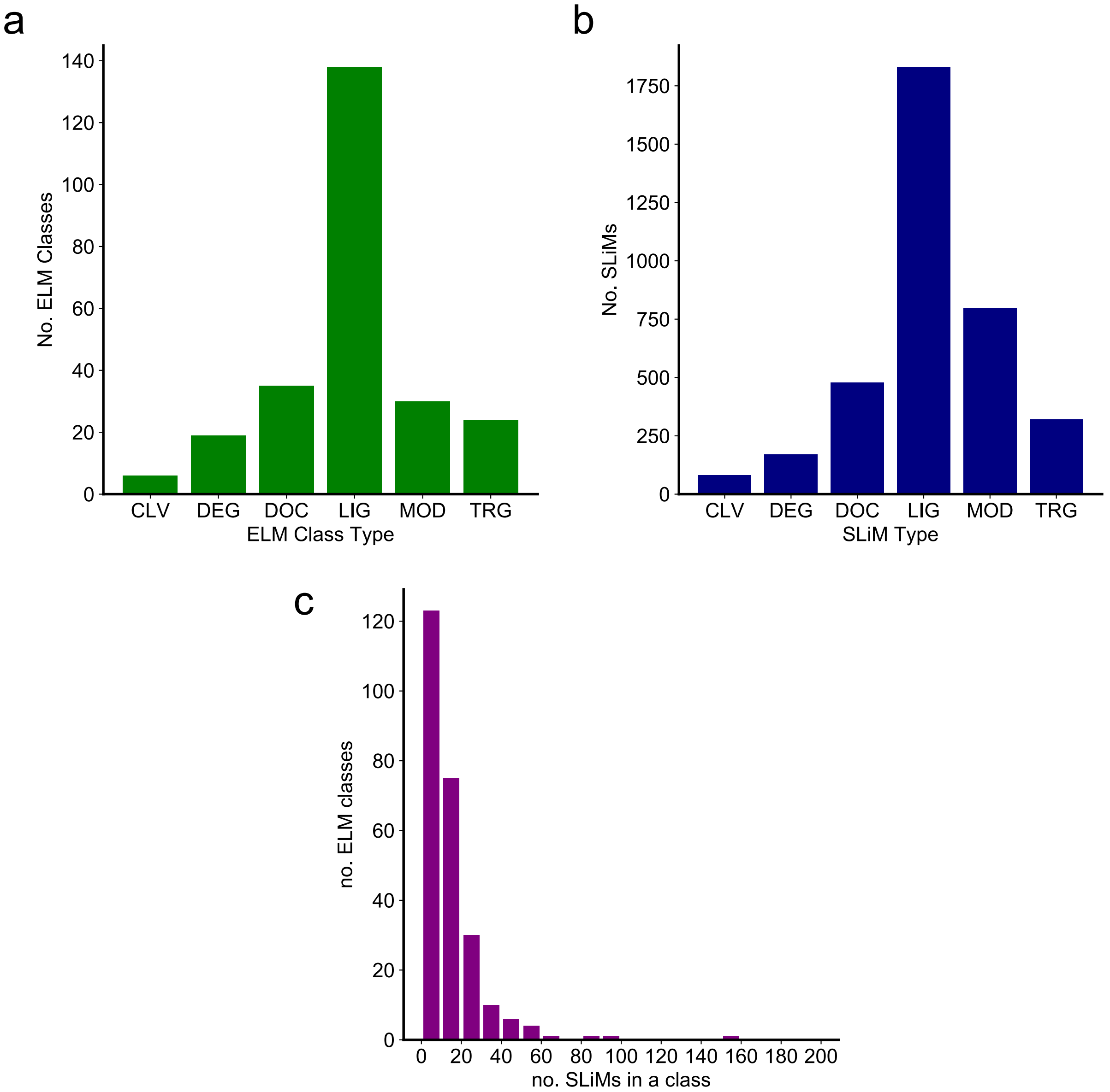

Figure S3. Distribution of the ELM benchmarking dataset by ELM-defined types. Distribution of the benchmarking dataset in terms of the number of ELM classes (a) and ELM instances/SLiMs (b), and the number of SLiMs in each ELM class (c).

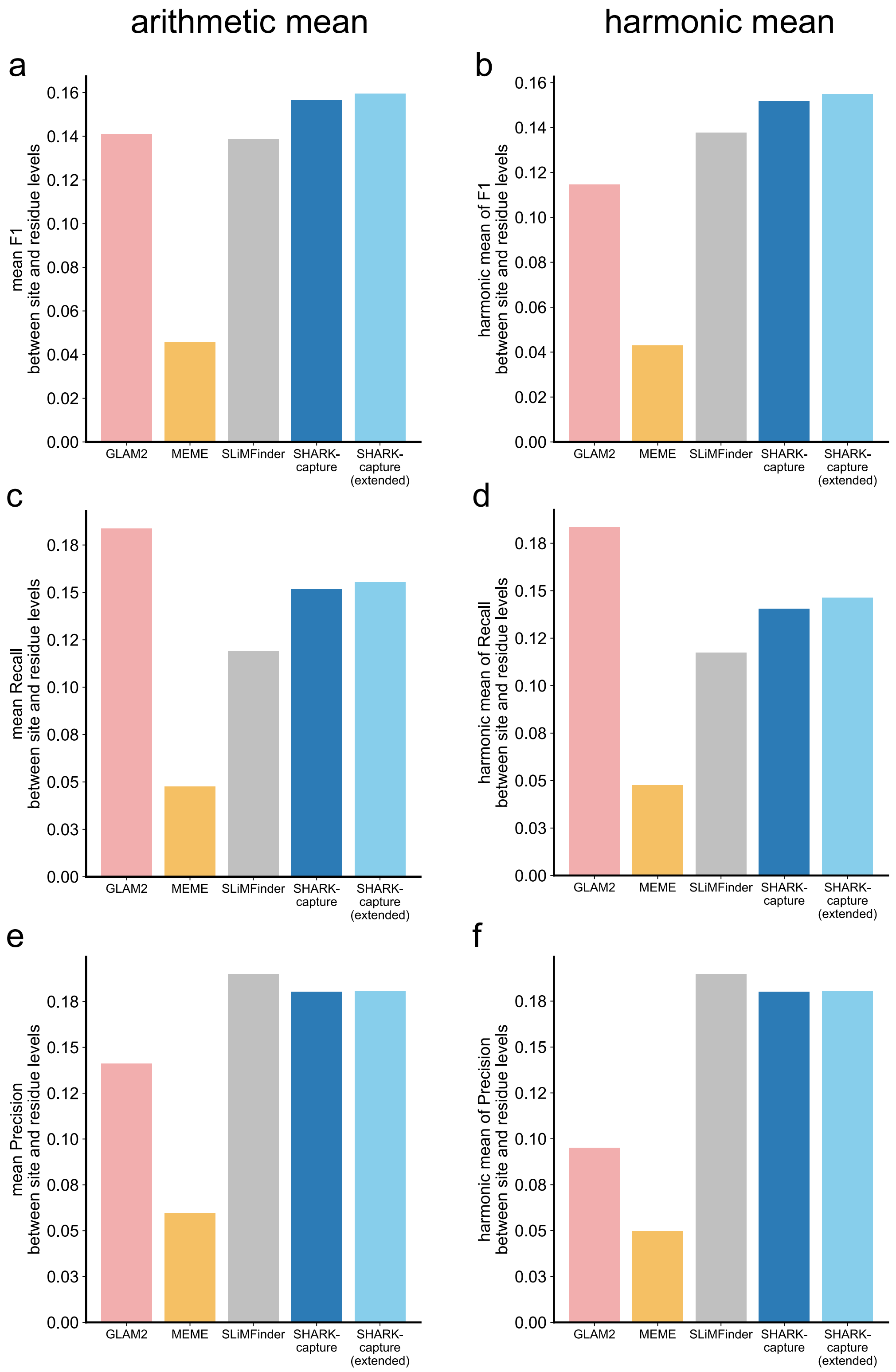

Figure S4. SHARK-capture offers best-in-class overall performance across a systematic ELM benchmark across site and residue levels. Arithmetic (a, c, e respectively) and harmonic (b, d, f) means of F1, recall and precision metrics across site and residue levels. The harmonic mean is calculated for completeness, since these metrics represent ratios of performance.

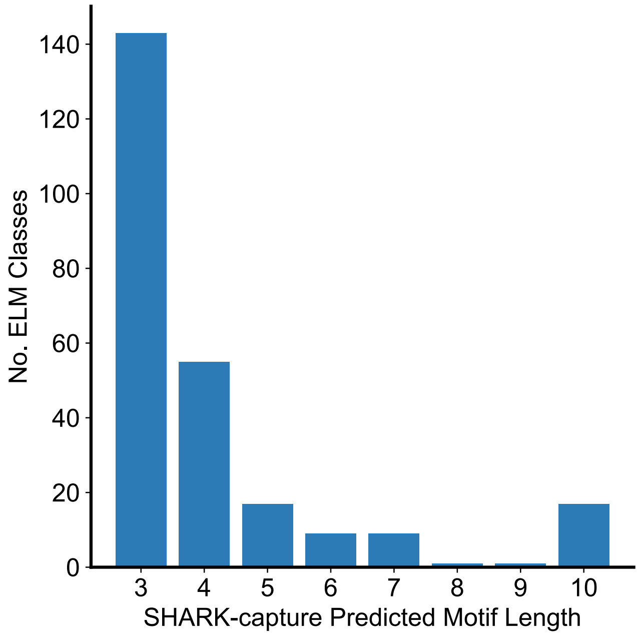

Figure S5. SHARK-capture predicted consensus *k*-mers show a broad distribution of lengths from k_{min} to k_{max} (3 and 10 respectively). SHARK-capture predicted consensus *k*-mer/motif lengths for all 252 ELM classes in the benchmark.

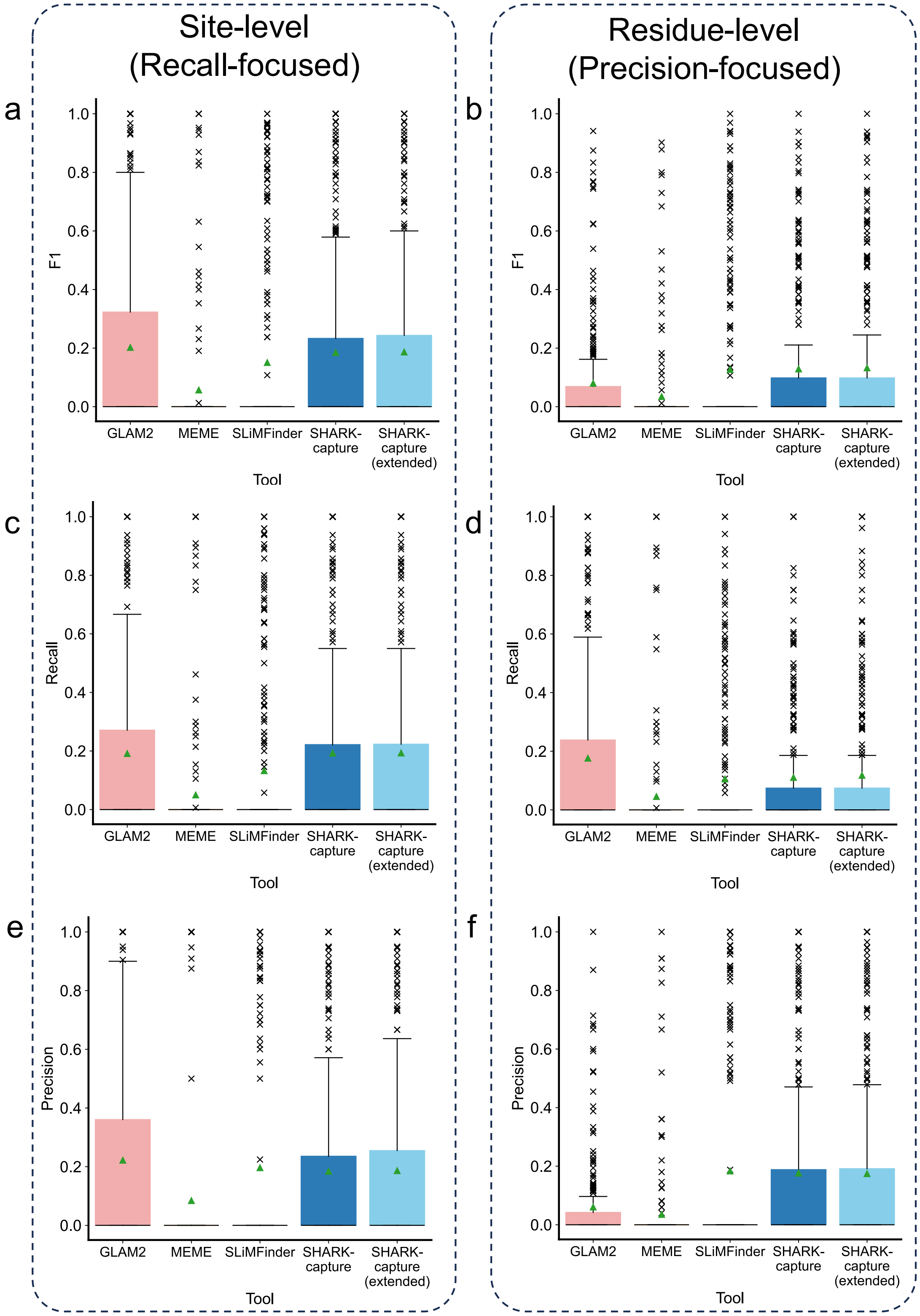

Figure S6. SHARK-capture offers capable performance across a systematic ELM benchmark. Boxplot distribution of the respective F1, Recall and Precision performance of each tool in the ELM benchmark. Due to the high number of classes where recall (and hence precision) is 0 (particularly for MEME and SLiMFinder), the (arithmetic) mean is also shown as a green triangle and summarized in S6. Outliers are shown as ‘x’.

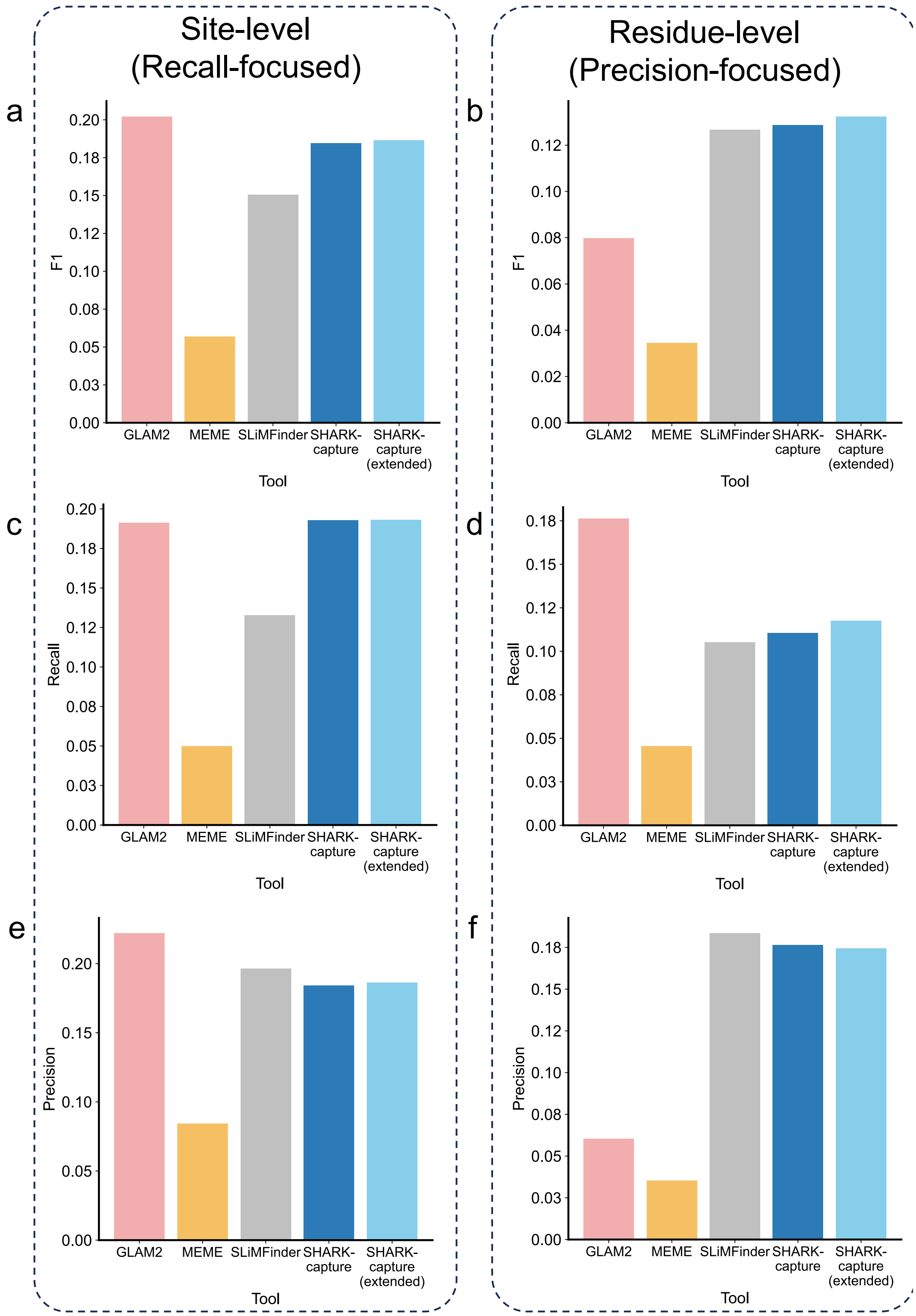

Figure S7. SHARK-capture offers capable performance across a systematic ELM benchmark. SHARK-capture offers strong site-specific recall (c, best-in-class) and residue-specific precision (f), resulting in strong residue-level (b, best-in-class) and site-level (a) overall performance as indicated by F1 score. For completion, site-specific precision (e) and residue-specific recall (d) values are also shown.

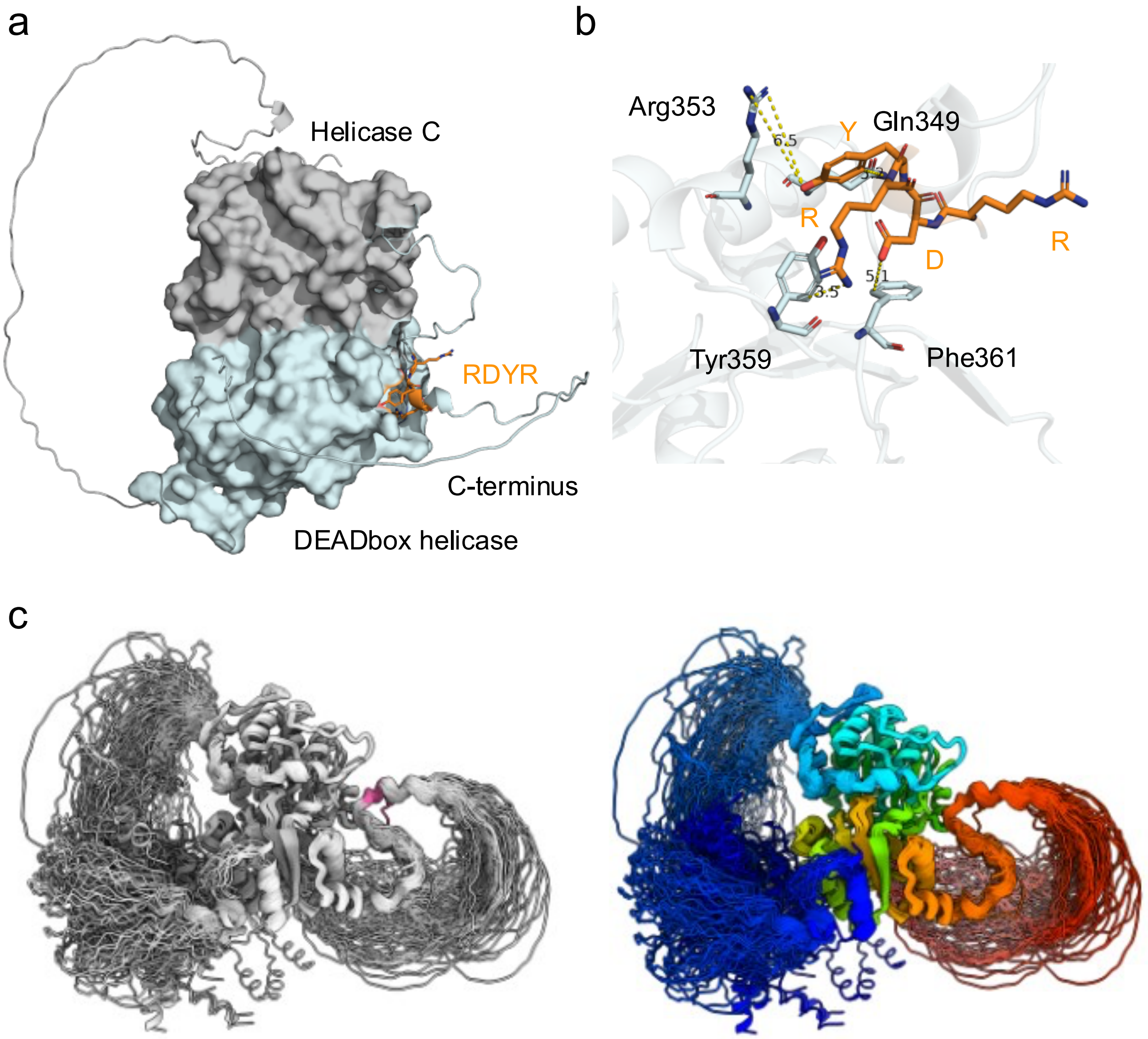

Figure S8. AlphaFold2 predicts RDYR motif-helicase core interactions. a) The RDYR motif (in orange) lies in close proximity with the helicase core, with multiple residues (b) lying in close proximity (<7Å) with helicase core (a.a. 99-535) residues. c) AlphaFold2 and 3 were run with 10 different seeds respectively to check for randomness of AlphaFold IDR prediction. Shown is the structural alignment of all 100 generated models, colored by residue number of the model (N-term blue, C-term red) or with the motif highlighted. Apart from one, all models fold the motif back onto the structured domain.

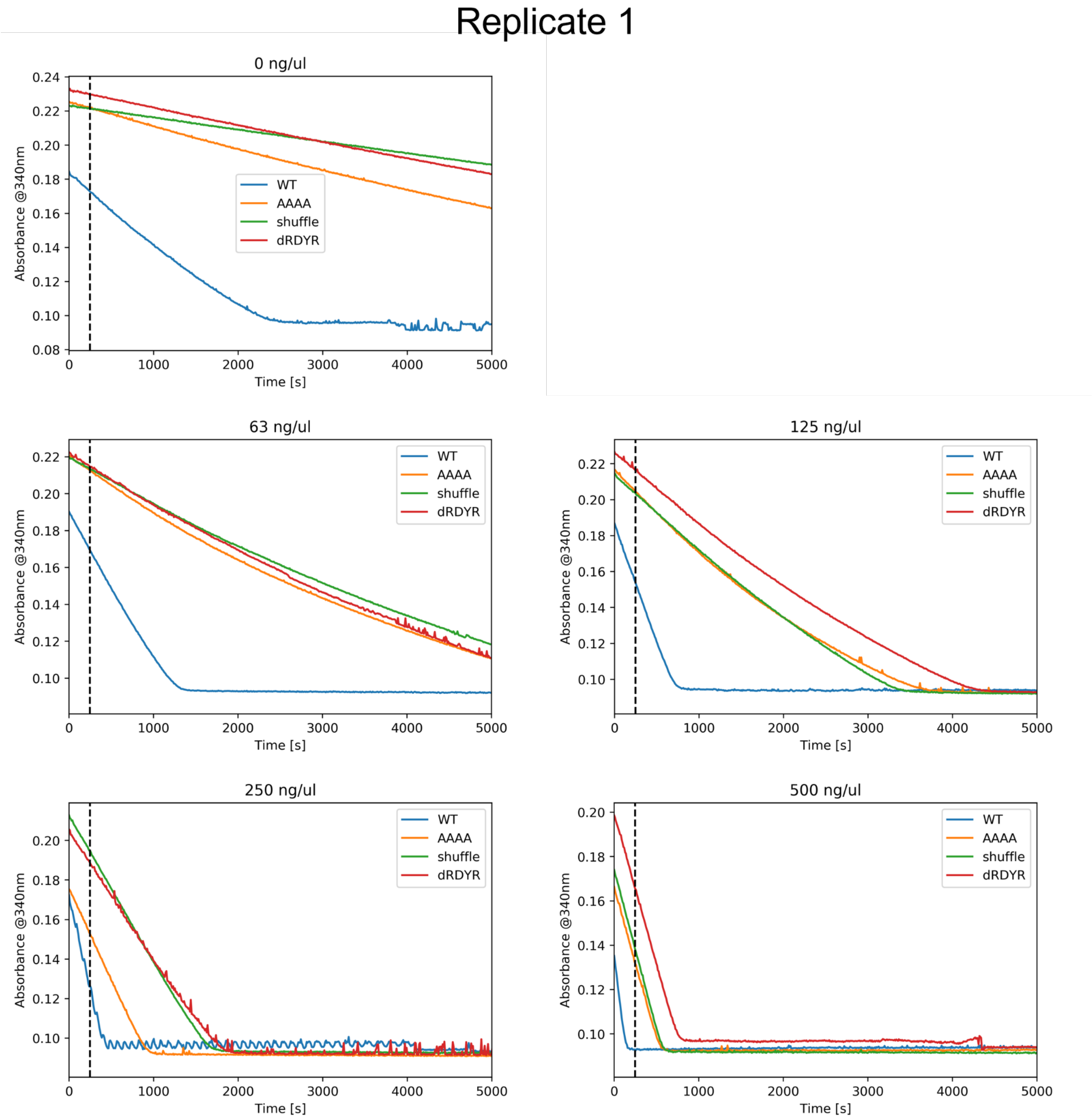

Figure S9. Absorbance of various Ded1p mutants in ATPase assay replicate 1. RNA concentration is stated in the title. Note that for this replicate there is no [RNA]=31 ng/μL. Hydrolysis rate is calculated between t=0 s and t=248 s (dotted line).

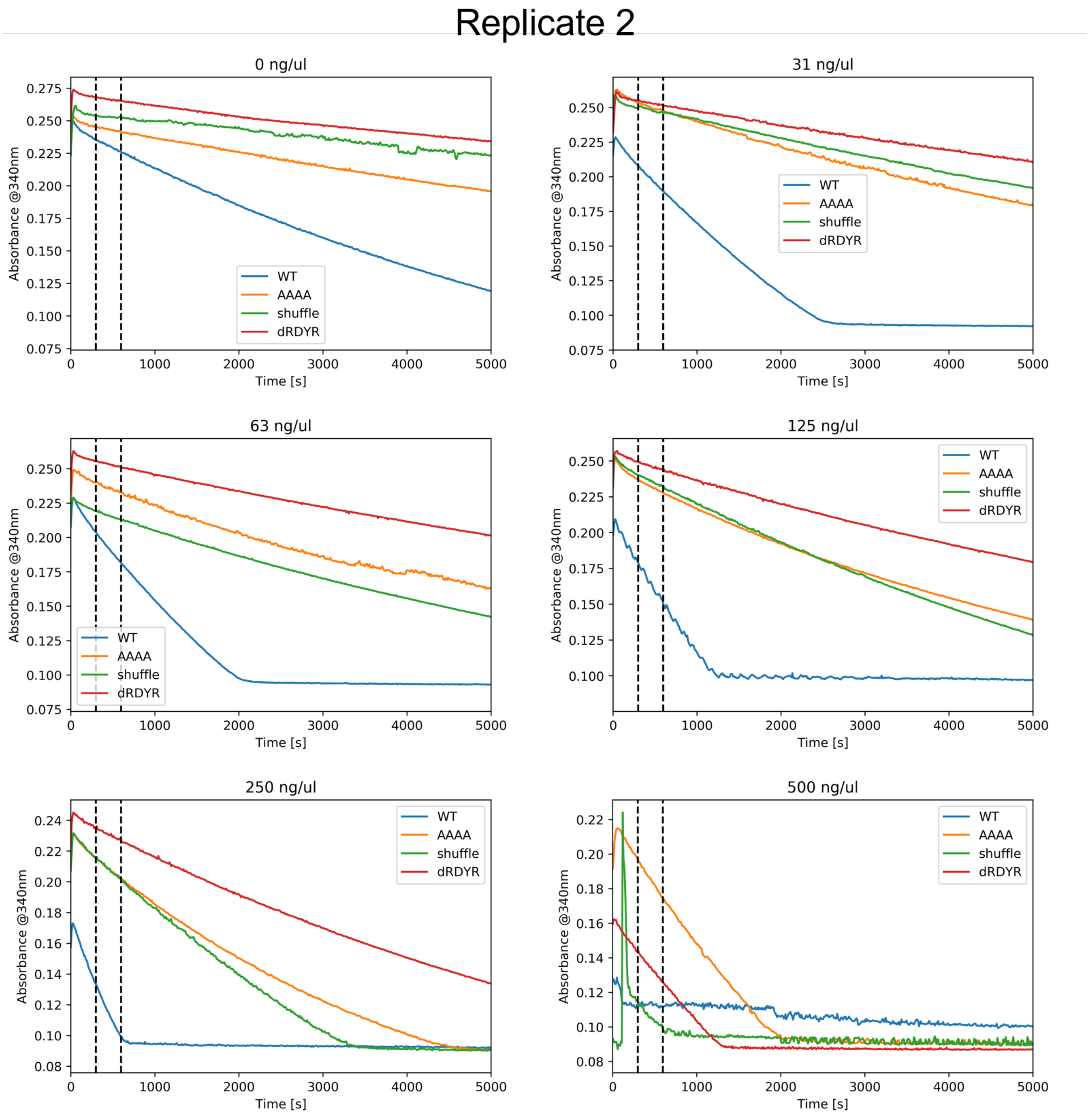

Figure S10. Absorbance of various Ded1p mutants in ATPase assay replicate 2. RNA concentration is stated in the title. Hydrolysis rate is calculated between t=297 s and t=595 s (dotted lines).

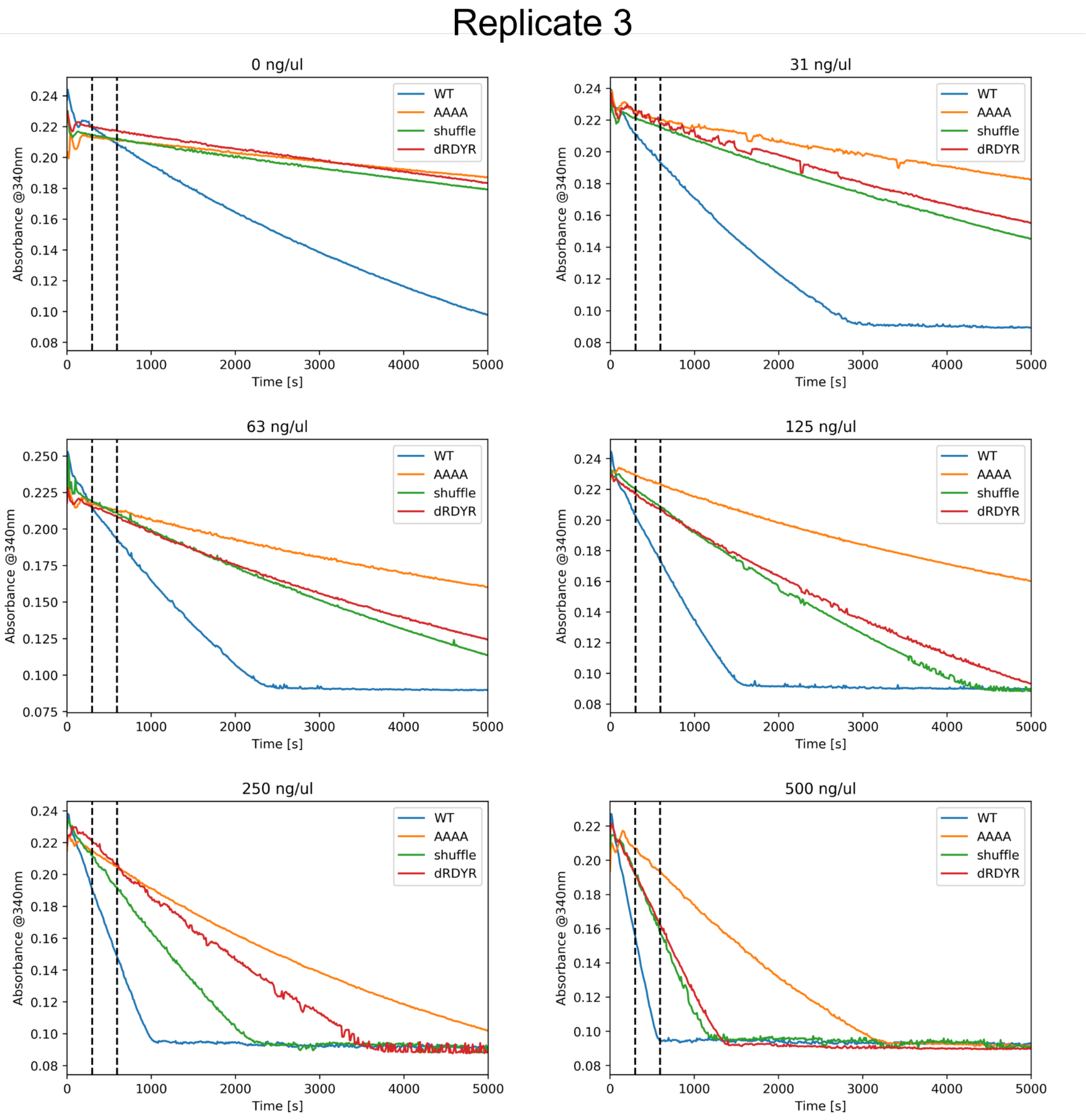

Figure S11. Absorbance of various Ded1p mutants in ATPase assay replicate 3. RNA concentration is stated in the title. Hydrolysis rate is calculated between t=297 s and t=595 s (dotted lines).

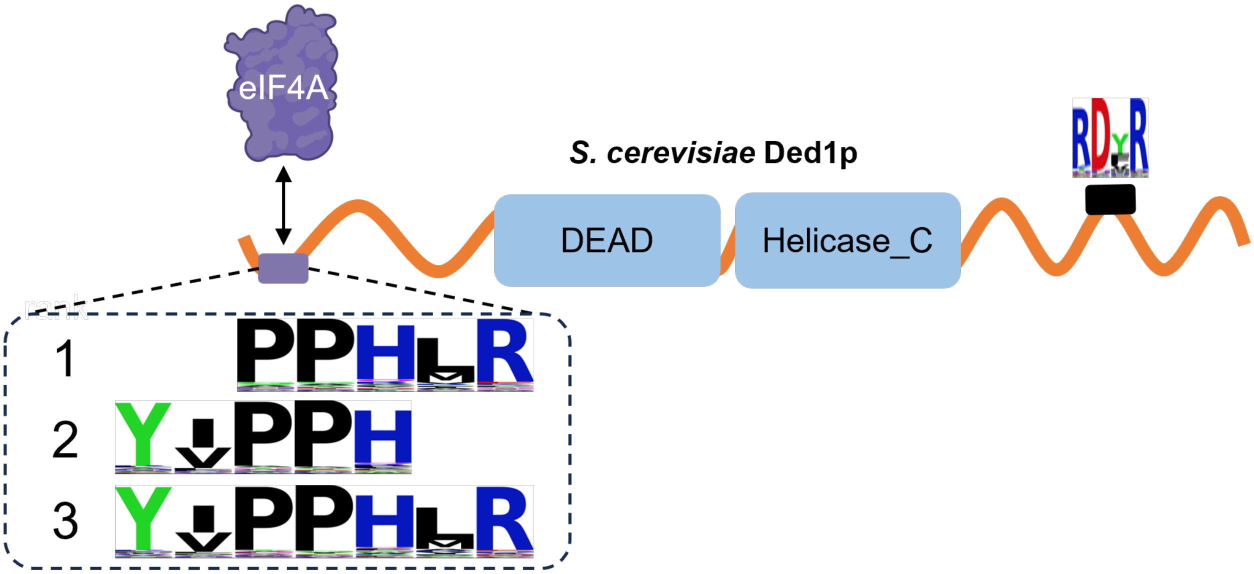

Figure S12. SHARK-capture identifies a highly conserved eIF4A interaction motif in the Ded1p N-terminus. The highly conserved V[I/V]PPHLR motif constitute the top 3 predictions by SHARK-capture.

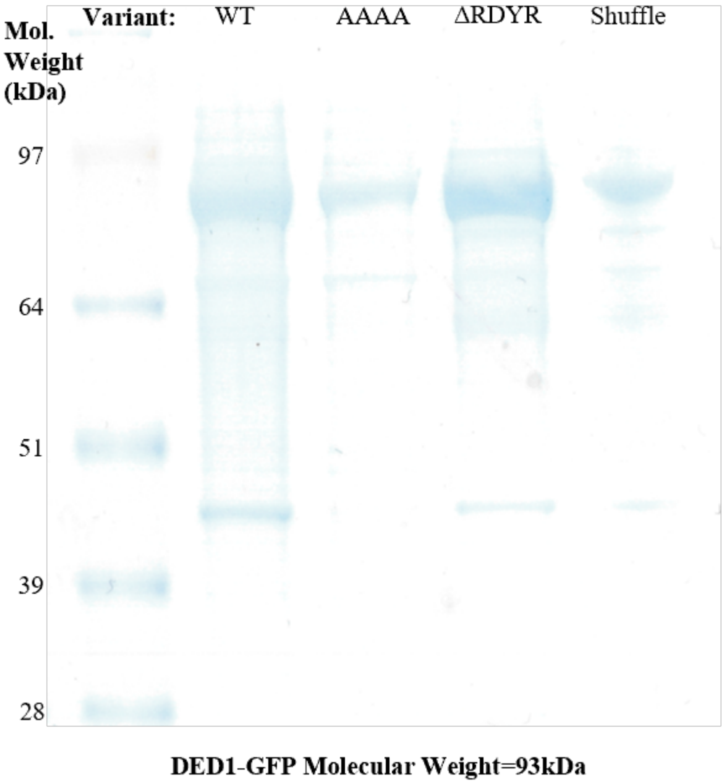

Figure S13. SDS-PAGE gel of purified Ded1p WT and mutants. As expected, the molecular weight from the SDS-PAGE is consistent with the expected weight of Ded1p+GFP (without MBP tag).

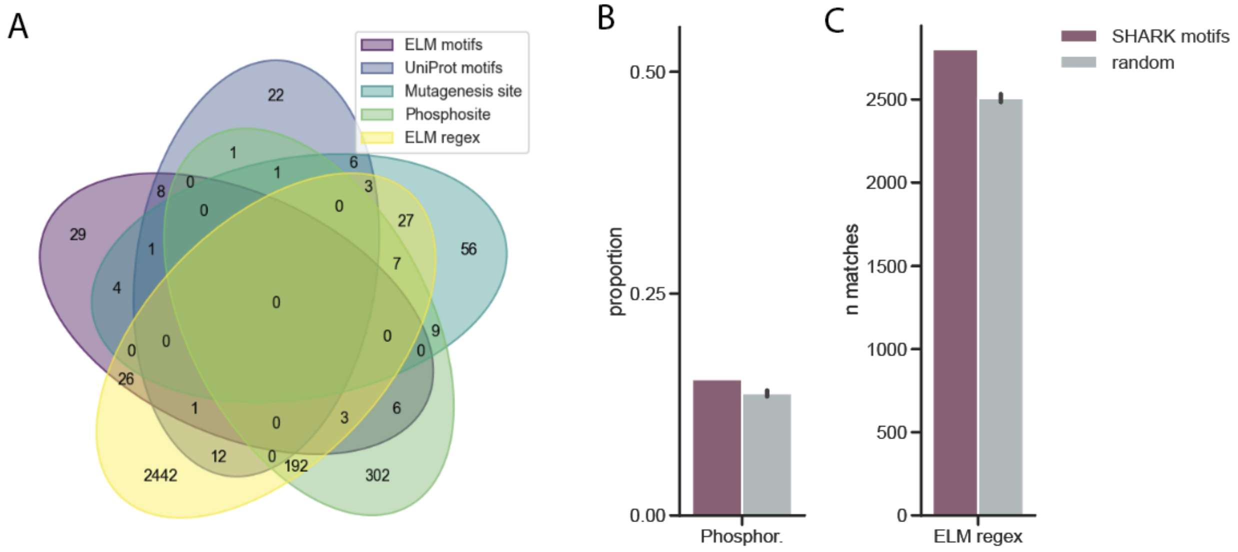

Figure S14. Annotations of proteome wide SHARK-capture predictions. A) Venn diagram showing the overlap of annotations of identified motifs. Comparison of SHARK-motifs matching phosphorylation sites (B, not significant by chi-square test) and ELM regex (C, p-value < 10^{-5} by chi-square test) to random control.

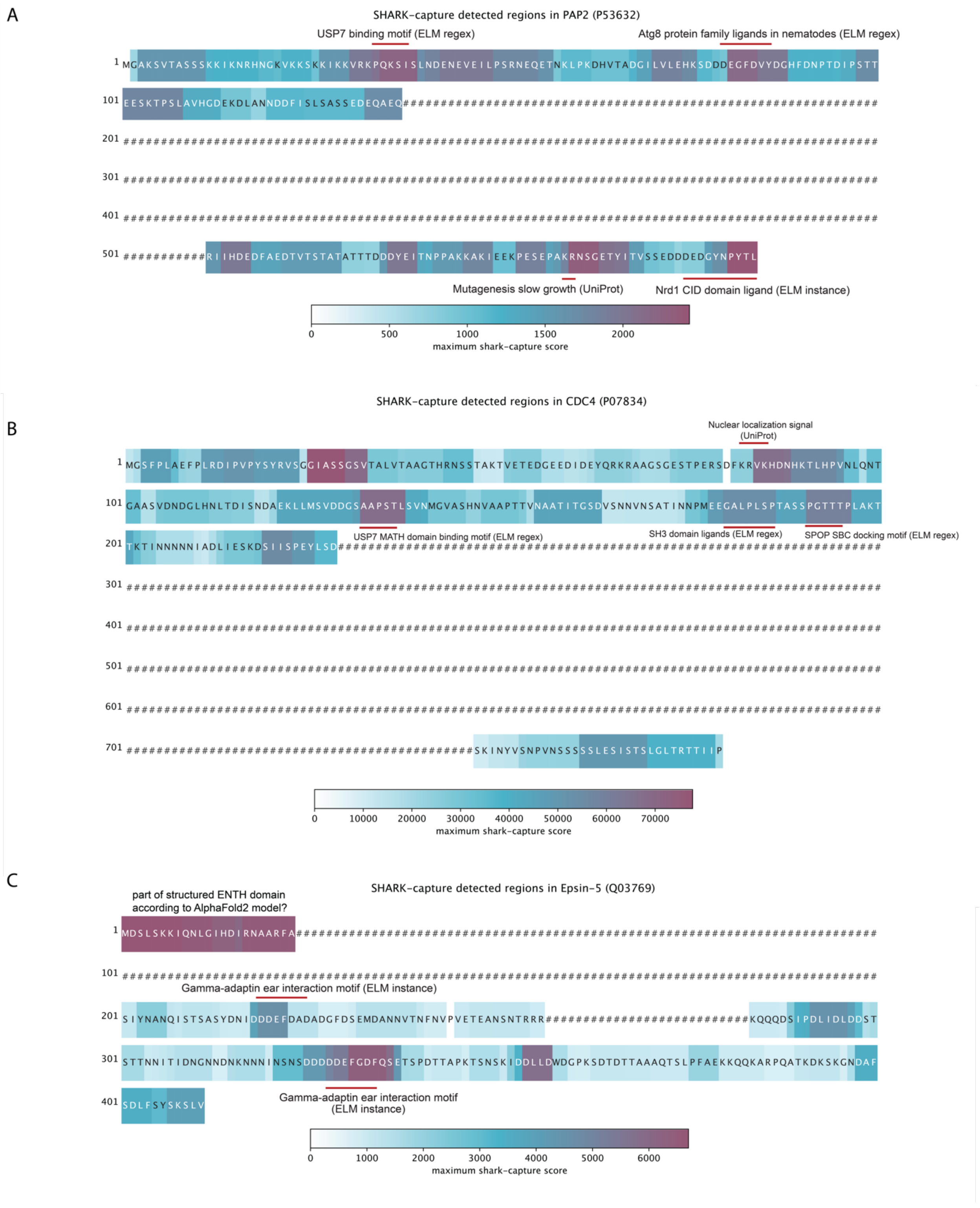

Figure S15. SHARK-capture score plots and annotations for A) PAP2 B) CDC4 and C) Epsin-5.

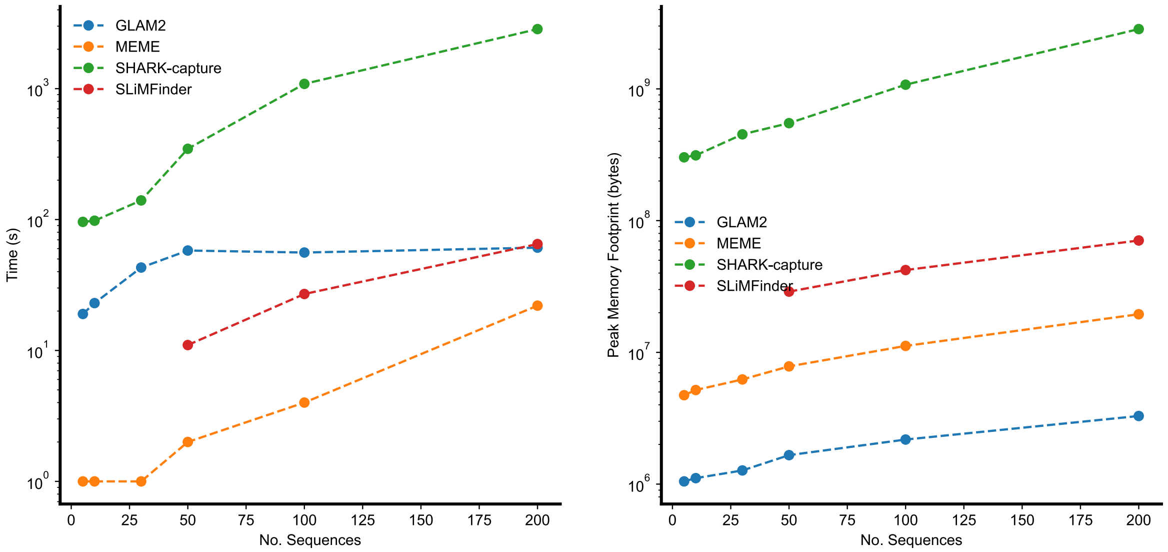

Figure S16. Time and Memory requirements for SHARK-capture and benchmarked motif detection tools. Note: Since MEME is run with default parameters, it returns only 1 detected motif, whilst SLiMFinder did not detect any motifs across all these runs.

Supplementary Tables

Tool	Sequences	Time (seconds)	Peak Memory Footprint (bytes)	Errors
MEME	5	1	4730880	
MEME	10	1	5177344	
MEME	30	1	6230016	
MEME	50	2	7827456	
MEME	100	4	11194348	
MEME	200	22	19472384	
GLAM2	5	19	1048576	
GLAM2	10	23	1110016	
GLAM2	30	43	1269760	
GLAM2	50	58	1658880	
GLAM2	100	56	2179072	
GLAM2	200	61	3289088	
SLiMFinder	5	2	14860288	Aborted, insufficient no. of UPCs
SLiMFinder	10	3	15040512	Aborted, insufficient no. of UPCs
SLiMFinder	30	2	16351232	Aborted, insufficient no. of UPCs
SLiMFinder	50	11	28909568	
SLiMFinder	100	27	42150632	
SLiMFinder	200	65	70668288	
SHARK-capture	5	96	302370816	
SHARK-capture	10	98	312995840	
SHARK-capture	30	140	451837952	
SHARK-capture	50	347	549429248	
SHARK-capture	100	1087	1075834880	
SHARK-capture	200	2850	2843648000	

Table S1. Time and Memory requirements for SHARK-capture and benchmarked motif detection tools.

Supplementary Datasets

Information on the benchmarking datasets and results are available as Supplementary Datasets S1-4 at https://doi.org/10.17617/3.TGOQYO, which includes:

Dataset S1. ELM benchmark information and outputs (Data_S1_ELM_benchmark.tar.gz)

Dataset S2. BuGZ input sequences and tool outputs (Data_S2_BuGZ.tar.gz)

Dataset S3. Ded1p input sequences, ATPase Assay Results and tool outputs (Data_S3_Ded1p.tar.gz)

Dataset S4. Motif prediction resource for *S. cerevisiae* proteome (Data_S4_yeast_motifs.tar.gz)

Supplementary References

1. Jegers C, Franzmann TM, Hübner J, Schneider J, Landerer C, Wittmann S, Toth-Petroczy A, Sprangers R, Hyman AA, Alberti S (2022) A conserved and tunable mechanism for the temperature-controlled condensation of the translation factor Ded1p. bioRxiv [Internet]:2022.10.11.511767. Available from: https://www.biorxiv.org/content/10.1101/2022.10.11.511767v1

2. Iserman C, Desroches Altamirano C, Jegers C, Friedrich U, Zarin T, Fritsch AW, Mittasch M, Domingues A, Hersemann L, Jahnel M, et al. (2020) Condensation of Ded1p Promotes a Translational Switch from Housekeeping to Stress Protein Production. Cell 181:818-831.e19.

3. Lemaitre RP, Bogdanova A, Borgonovo B, Woodruff JB, Drechsel DN (2019) FlexiBAC: a versatile, open-source baculovirus vector system for protein expression, secretion, and proteolytic processing. BMC Biotechnol. 19:20.
